# Horizontally transferred genes in the genome of Pacific white shrimp, *Litopenaeus vannamei*

**DOI:** 10.1186/1471-2148-13-165

**Published:** 2013-08-06

**Authors:** Jian-Bo Yuan, Xiao-Jun Zhang, Cheng-Zhang Liu, Jian-Kai Wei, Fu-Hua Li, Jian-Hai Xiang

**Affiliations:** 1 Key Laboratory of Experimental Marine Biology, Institute of Oceanology, Chinese Academy of Sciences, 7, Nanhai Road, Qingdao 266071, China; 2 University of Chinese Academy of Sciences, Beijing 100049, China

**Keywords:** Horizontal gene transfer, *Litopenaeus vannamei*, Shrimp, Bacteria, Fungi

## Abstract

**Background:**

In recent years, as the development of next-generation sequencing technology, a growing number of genes have been reported as being horizontally transferred from prokaryotes to eukaryotes, most of them involving arthropods. As a member of the phylum Arthropoda, the Pacific white shrimp *Litopenaeus vannamei* has to adapt to the complex water environments with various symbiotic or parasitic microorganisms, which provide a platform for horizontal gene transfer (HGT).

**Results:**

In this study, we analyzed the genome-wide HGT events in *L. vannamei*. Through homology search and phylogenetic analysis, followed by experimental PCR confirmation, 14 genes with HGT event were identified: 12 of them were transferred from bacteria and two from fungi. Structure analysis of these genes showed that the introns of the two fungi-originated genes were substituted by shrimp DNA fragment, two genes transferred from bacteria had shrimp specific introns inserted in them. Furthermore, around other three bacteria-originated genes, there were three large DNA segments inserted into the shrimp genome. One segment was a transposon that fully transferred, and the other two segments contained only coding regions of bacteria. Functional prediction of these 14 genes showed that 6 of them might be related to energy metabolism, and 4 others related to defense of the organism.

**Conclusions:**

HGT events from bacteria or fungi were happened in the genome of *L. vannamei*, and these horizontally transferred genes can be transcribed in shrimp. This is the first time to report the existence of horizontally transferred genes in shrimp. Importantly, most of these genes are exposed to a negative selection pressure and appeared to be functional.

## Background

Unlike vertical transfer, horizontal gene transfer (HGT) refers to the transfer of genes between organisms and allows recipients to acquire novel traits from the donors [1]. In most cases, horizontally transferred genes (HGT genes) are nonfunctional. However, functional HGTs often benefit the recipients in terms of adaptation to certain specialized niches and are thought to be an important evolutionary impetus [2]. Nowadays, most detected HGT events occur among bacteria inhabiting the same environment, and are a major source of genetic variation in bacteria [3,4]. However, HGT is also increasingly acknowledged to play an important role in animals [2]. Predominantly prokaryotes-to-animal HGT events have been described between prokaryotes and arthropods or nematodes, and an endosymbiont is the most probable donor for gene transfer because of the close and constant proximity of the cells from both organisms [1,5-9]. HGT in animals causes divergence in genetic materials and may result in physiological metabolism improvements and the gain of some other phenotypes, e.g. virulence gene transfer can cause emergence of new disease, while HGT genes in aphids can help it produce carotenoid [10-13]. Furthermore, functional HGT genes in animals have been identified play a vital role in the evolution of recipients [1,2].

The Pacific white shrimp, *Litopenaeus vannamei*, is an economically important marine aquaculture species worldwide. However, the development of the commercial culture of shrimps has generally been accompanied by increasing problems with diseases, which are mostly caused by opportunistic pathogens, such as viruses, bacteria and fungi [14]. Erosions of the cuticle, localized lesions and generalized septicemias are the three general symptoms seen in infected shrimps [14]. Generally, shrimps living in complex environments with various parasitic or symbiotic microorganisms, and the communications between them always make effects on their growth [15-18]. The close relationship between shrimps and their parasitic or symbiotic organisms may provide a platform for trans-kingdom horizontal transfer of genetic materials during evolutionary history [1,3]. However, no strong evidence for the HGT events in shrimp has been found.

The detection of animal HGT events has been based on complete genome sequences and the combination of homology searching and phylogenetic analysis [19]. Unfortunately, the whole genome and complete gene set of shrimp have not been published. However, recently, a large number of expressed sequences tags (ESTs) and high-throughput transcriptome sequencing data of *L. vannamei* were published [20,21]. These data provide good resources for the exhaustive HGTs detection in shrimp. In this study, the candidate HGT genes in the genome of *L. vannamei* were searched by sequence homology comparison, phylogenetic analysis, and experimental confirmation of the candidates HGT genes. By comparing the HGT genes-located shrimp genome contigs and corresponding donor genomes, the relatively large genomic segments, which included gene clusters, have been identified as being horizontally transferred from bacteria and integrated into the shrimp genome. Furthermore, their expression profiles at five developmental stages of shrimp were analyzed and their probable functions in shrimp were discussed.

## Results

### Fourteen HGT genes were detected in *L. vannamei*

**Figure 1:**
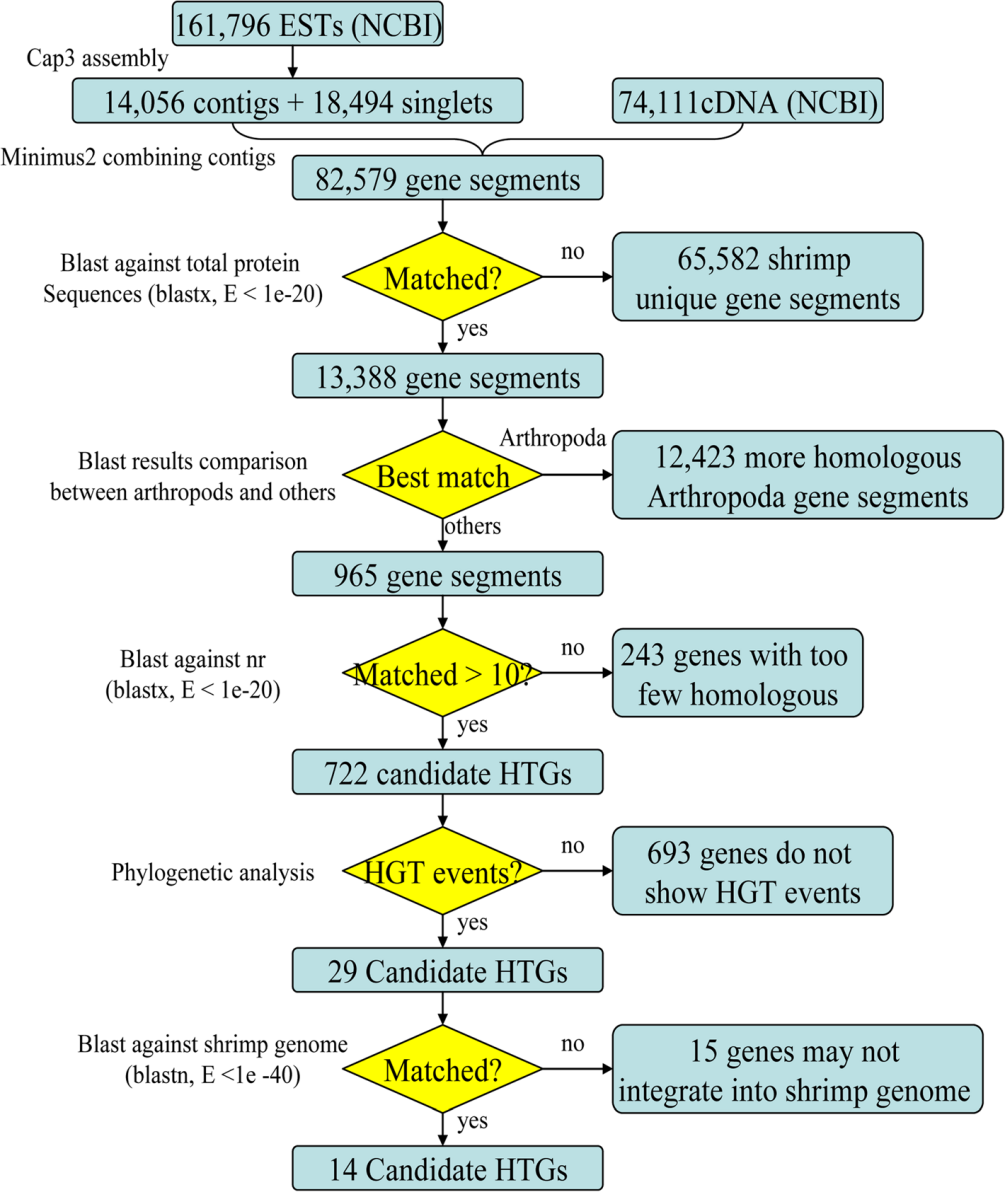
**Flowchart for identification of HGT genes in *L. vannamei*.** The eliminated and remaining numbers of gene segments in each step are listed on the figure.

In this study, an exhaustive detection method was used to identify HGT genes in *L. vannamei* (see Methods). Homologous BLAST analysis was used initially to detect HGT genes with a view for identifying homologous genes of non-mating species from shrimp (Figure 1). Initially, 65,582 gene segments were filtered out because there were no homologs detected among them. These sequences probably represent shrimp unique gene segments or non-coding DNA. In the second stage of homologous searching, more than 92% of the gene segments, which were most similar to other arthropod sequence, were excluded. Then, only 965 HGT candidates were left. During this procedure, two classes of sequences were filtered out: gene segments that only showed homology to arthropods and those having higher BLAST similarity scores with arthropods than any other species. However, several potential HGT events may be missed by this procedure, in that some truly HGT of Arthropoda may be considered as vertical inheritance. In the third homologous searching procedure, BLAST searches against the GenBank non-redundant protein database (nr) were implemented to extract sequences from an even larger spectrum of species. As small numbers of homologs are not sufficient for phylogenetic analysis and tree construction, we selected HGT candidates with more than 10 homologs in the nr database. Thus, after filtering genes in above procedures, only 722 HGT candidates remained for phylogenetic analysis (Figure 1).

Three kinds of phyloenetic analysis were performed on the remaining 722 HGT candidates after homology search (see Methods). Phylogenetic trees for each candidate HGT gene were constructed manually, and once a gene was nested within a donor clade and many other donor species formed basal branches, it was considered to be a HGT gene that had horizontally transferred from the donor to *L. vannamei*[8,22]. Most of the HGT candidates showed higher similarity with Arthropoda-related species and grouped within a single clade. Furthermore, several candidate HGTs showed a phylogenetic topology that did not support a HGT event. Thus, as for those 722 HGT candidates, 693 of them were eliminated by the phylogenetic analysis, leaving only 29 genes that displayed rational HGT phylogenetic topology (Figure 1). They were subjected to comparative phylogenetic analysis by constructing three kinds of phylogenetic trees. Ultimately, well-refined maximum likelihood (ML) trees were used to represent the HGT events.

Among the 29 candidate HGT genes above after phylogenetic selection, there were 12 genes showed significant similarity to the best-hit sequences (E-values ranged from 2.00E-82 to 5.00E-36, and identity values ranged from 95.83% to 100%). Considering that many candidate HGT genes might be contaminants derived from RNA extraction, library construction or sequencing, these sequences needed to be excluded from the 29 candidate HGT genes. Previously, we acquired 42× coverage of shotgun reads generated from whole genome sequencing of *L. vannamei*. A 1.9 Gb draft map of the contigs was assembled and used to identify genuine shrimp gene segments. Ultimately, only 14 candidate HGT genes were homologous to shrimp genomic contigs (E-values ranged from 0 to 9.00E-21, and identity values ranged from 88.22% to 100%). Thus, there are 15 genes were considered as non-shrimp genes and removed in this procedure. Some of HGT genes may be missed because of incompletely contigs assembly, but the 42× coverage of reads were full enough for identification of the existence of these gene segments. Generally, the sequenced reads tend to equally cover the assembly contigs, and eukaryotic contigs were expected to have different reads coverage to those contaminating contigs. Thus, with the help of SOAPaligner (http://soap.genomics.org.cn/soapaligner.html), we aligned all the reads to the 14 contigs which showed homologous with the 14 candidate HGT genes. The alignment results were compared with that of 1,000 randomly selected assembly contigs. It was found that the reads coverage of the 14 contigs (average coverage of 37.42) did not display any significant bias with that of 1,000 randomly selected contigs (average coverage of 38.25, Student’s *t*-test p < 0.05). Therefore, the 14 contigs seemed to possess eukaryotic characteristics rather than contaminates.

To avoid artificial mis-assembly of genomic contigs, PCR amplification was performed for these candidate HGT genes using shrimp genomic DNA as template. As expected, all 14 candidate HGT genes were successfully amplified*.* These amplified fragments were sequenced and showed nearly 100% sequence identity with correspondent genomic contigs. Furthermore, in order to verify the 14 HGT genes were integrated into the genome of *L. vannamei*, PCR amplification was also performed on both sides of the 14 genomic contigs on which the 14 HGT genes located. The two sides of the genome contigs, that did not show any similarity with the genome of the most probable donor, were most likely the eukaryotic sequences rather than HGT origin. BLAST results of the two sides of HGT fragments against nt database showed that most of them are similar to eukaryotic sequences (Additional file 1: Table S3). It indicates that the sides of these HGT fragments are eukaryotic portion, while the middle of the contigs is most probable HGT regions. Thus, we amplify the eukaryotic portion as long as possible to make sure the PCR products containing both eukaryotic and HGT regions. From the results, we can see that most of the edge products have been successfully amplified except for that of *rpsN*, which displayed a relatively weak amplification compared with the others. All the products were fully sequenced, and they were all identical to the correspondent regions of genome contigs. For the control of PCR amplification, all the products of positive control has been successfully amplified, while the products of negative control were amplified with nothing (see Methods section), which support for accuracy of the amplification of our target products. From the results above, it is reasonable to confirm that the 14 HGT genes were integrated into shrimp genome. In Table 1, the assigned names of the 14 HGT genes were based on their annotated functions or the gene name registered in NCBI (*acsf*, *rpsF*, *rpsN*, *exbB*, *mopB*, *tnpA*, *stat*, *rpc2*, *dhfr*, *cata*, *sdrp*, *omtp*, *ankp* and *deha*).

**Table 1:** **Fourteen predicted HGT genes in the *L. vannamei *genome**

**Symbol**	**Length**	**Types**^**a**^	**Contig position**	**GenBank ID**	**Function**	**Top hit species**^**b**^	**Phylum**	**Accession GI**	**E-value**	**Identity**	**Coverage**	**Figures**
*acsf*	672	B → L	Contig124858619: 312-944	KC701594	acetyl-coenzyme A synthetase family protein	*Desulfotomaculum kuznetsovii*	Chloroflexi	667983253	5.00E-48	66.87	72.77%	Figure S1
*rpsF*	395	B → L	Contig124943021: 4082-4445	KC701595	30S ribosomal protein S6	*Flavobacteriales bacterium*	Bacteroidetes	1163788026	6.00E-50	96.61	44.81%	Figure S2
*rpsN*	593	B → L	Contig5551: 3683-4206	KC701598	50S ribosomal protein L21	*Gramella forsetii*	Bacteroidetes	1160887756	2.00E-58	79.31	88.03%	Figure S3
*exbB*	522	B → L	Contig124942315: 3448-3593	KC701596	biopolymer transport protein	*Robiginitalea biformata*	Bacteroidetes	3320593070	1.00E-78	96.89	92.53%	Figure S4
*mopB*	760	B → L	Contig124944249: 1863-1980	KC701597	molybdopterin oxidoreductase, iron-sulfur binding subunit	*Polaribacter irgensii*	Firmicutes	3336447549	5.00E-123	83.27	99.08%	Figure S5
*tnpA*	352	B → L	Contig5540: 2118-2468	KC701599	transposase	*Escherichia Coli*	Gama-proteobacteria	269961834	1.00E-60	100	98.01%	Figure S6
*stat*	784	B → L	Contig124277025: 193-313	KC701600	streptomycin 3″-adenylyltransferase	*Shigella flexneri*	Gama-proteobacteria	3331678336	8.00E-78	100	56.25%	Figure S7
*rpc2*	778	B → L	Contig124717565: 157-356	KC701601	repressor protein C2	*Escherichia Coli*	Gama-proteobacteria	3333019710	6.00E-47	94.74	21.98%	Figure S8
*dhfr*	692	B → L	Contig124922462: 426-1117	KC701602	dihydrofolate reductase	*Vibrio harveyi*	Gama-proteobacteria	3323184048	2.00E-52	69.57	69.80%	Figure S9
*cata*	709	B → L	Contig147675: 106-530	KC701603	chloramphenicol acetyltransferase	*Bacteroides uniformis*	Bacteroidetes	1120437905	1.00E-81	100	59.24%	Figure S10
*sdrp*	286	B → L	Contig124657743: 33-616	KC701604	short-chain dehydrogenase/reductase SDR	*Burkholderia phymatum*	Beta-proteobacteria	1186471973	3.00E-29	67.37	99.99%	Figure S11
*omtp*	660	B → A	Contig123317449: 1-217	KC701605	O-methyltransferase family protein	*Francisella sp.*	Gama-proteobacteria	88802104	5.00E-23	44.65	72.27%	Figure 2
*ankp*	955	F → L	Contig124712867: 86-719	KC701606	ankyrin repeat-containing protein	*Grosmannia clavigera*	Ascomycota	2260060705	2.00E-25	46.39	30.47%	Figure S12
*deha*	761	F → L	Contig121079137: 1-172	KC701607	DEHA2A03014p	*Debaryomyces hansenii*	Ascomycota	294654414	8.00E-22	37.23	54.01%	Figure S13

^a^The HGT types of each HGT gene. B → L indicates HGT from Bacteria to *L. vannamei* or its ancestor; B → A indicates HGT from Bacteria-to-Arthropoda or its ancestor; F → L indicates HGT from Fungi-to-*L. vannamei* or its ancestor. ^b^The Arthropoda-related sequences were removed from the top hit BLAST species.

### Bacteria and fungi were the two predominating donors of HGT genes

The phylogenetic trees can be used to indicate HGT events and their directions [22,23]. For 11 of the 14 HGT genes, the phylogenetic trees showed a phylogenetic topology of *L. vannamei* nesting with bacteria but far from other eukaryotes, which indicated a bacteria-to-*L. vannamei* HGT event (Table 1, Additional file 2: Figures S1, S2, S3, S4, S5, S6, S7, S8, S9, S10 and S11). The most probable donors of bacteria-to-*L. vannamei* HGT genes were Proteobacteria (five species) and Bacterioidetes (four species), and most of them were ecologically related to shrimp. *Desulfotomaculum kuznetsovii*, *Flavobacteriales bacterium*, *Robiginitalea biformata*, *Polaribacter irgensii*, *Gramella forsetii*, and *Vibrio harveyi* are marine bacteria, while *Escherichia coli* and *Bacteroides uniformis* are gut bacteria. These results supported the view that HGT events generally occur between species inhabiting the same environment [1,3]. The other two HGT genes were transferred from fungi to *L. vannamei* (*ankp* and *deha*, Table 1). Phylogenetic trees of *ankp* and *deha* showed that *L. vannamei* completely nested within the clade of fungi and far from bacteria and other eukaryotes, indicating a transfer of fungal origin (Additional file 2: Figures: S12, S13). The best-hit species of these two HGT genes belong to the Ascomycota, which is the largest phylum of fungi [24]. One of the two fungi donors, *Grosmannia clavigera*, was reported to be a symbiont of the mountain pine beetle [19]. Few fungi-to-higher eukaryote HGT events have been detected in previous studies, whereas, some reports of fungi-to-higher eukaryote HGT genes indicated that they greatly improved the host’s phenotypes and evolution [12,25]. Subsequent functional analysis indicated that these two fungi originated HGT genes may function in protein-protein interactions and electron transfer, respectively.

**Figure 2:**
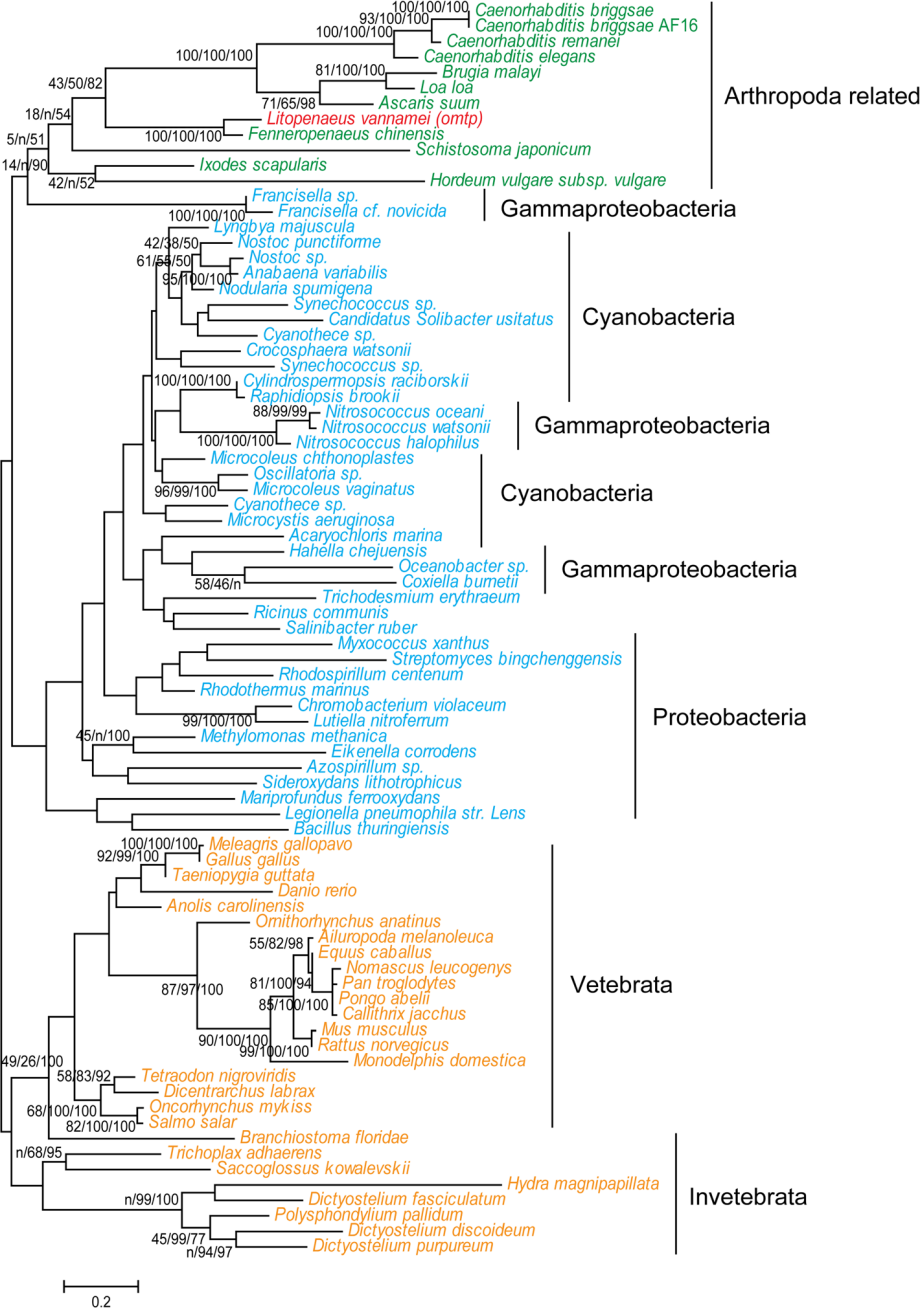
**Phylogenetic position of the shrimp HGT gene *omtp*.** The phylogenetic tree was constructed using Phyml to perform ML analysis. The support values of ML, NJ and BI analysis are displayed beside each node.

BLAST results of HGT genes against the nr database showed that the above 13 of the 14 HGT genes have no homologs among any other arthropods, which suggests that these HGT genes were transferred to *L. vannamei* or its ancestor after the speciation of Arthropoda [8]. One exception was a HGT gene, *omtp*, which showed high similarity with sequences of other Arthropoda-related species (arthropods and nematodes) that were all nested within the group of bacteria while being phylogenetically far from other invertebrates and vertebrates (Table 1, Figure 2). Previous phylogenetic analysis clustered arthropods and nematodes in a clade of molting animals termed the Ecdysozoa [26], which indicated they are evolved from the same ancestor. Besides, *omtp* was considered to encode a catechol-*O*-methyltransferase (COMT), a protein family which has ever been found to be horizontally transferred from bacteria to eukaryotes before the separation of animals and fungi [27,28]. This indicates that *omtp* was horizontally transferred earlier than the other HGT genes, and our phylogenetic tree of *omtp* suggested that it was transferred from a bacterial source to arthropods or its ancestors before the speciation of Ecdysozoa.

### Four HGT genes contain introns

Structure analysis of HGT genes was used to investigate whether there is structural evolvement of the HGT genes between the donor and recipient, which may provide evidence of the HGT events. Seven HGT genes were derived from single EST or transcriptome sequences: *tnpA* (GI: 41059456), *stat* (GI: 57504717), *rpc2* (GI: 57504772), *dhfr* (GI: 171573934), *cata* (GI: 259510079), *sdrp* (GI: 349908938), *deha* (GI: 171560878). When comparing HGT gene segments to the shrimp genome contigs, we found that four HGT genes (*acsf*, *sdrp*, *ankp* and *deha*) contained introns in the shrimp genome. *ankp* and *deha* are the two HGT genes derived from fungi and their homologous genes in fungal genomes also contain introns, whereas *acsf* and *sdrp* were transferred from bacteria, which were free of introns. Surprisingly, it was found that the introns of genes from the donor fungi were different from those in the genome of shrimp, whereas the coding regions around the introns were highly conserved (E-values ranged from 8.00E-17 to 2.00E-13). Besides, by alignment of shrimp genome sequencing reads to this gene using SOAPaligner, we found that the intron regions were in highly coverage of nearly 88X, which is significantly higher than the sequencing depth of the whole genome (42X). Furthermore, these two HGT genes are evenly covered at 76X, while the random selected 100 genes is covered at 48X. Through using SOAPsnp v1.03 [29], three SNPs have been found on the contigs on which *ankp* located, and one of them has been detected locate on the intron of *ankp*. This indicated that the introns in two fungi-originated HGT genes may be highly repeated along with its surrounding sequences in shrimp genome. The shrimp *acsf* contains two introns (105 bp and 117 bp in length, respectively) and *sdrp* contains one intron (370 bp in length), but no introns were detected in the corresponding donor bacteria genome (*D. kuznetsovii* and *B. phymatum*). Therefore, it can be speculated that the introns might be integrated or changed in the HGT genes after horizontal transfer from the donor to *L. vannamei*.

### Three large exogenous segments integrated into *L. vannamei* genome

**Figure 3:**
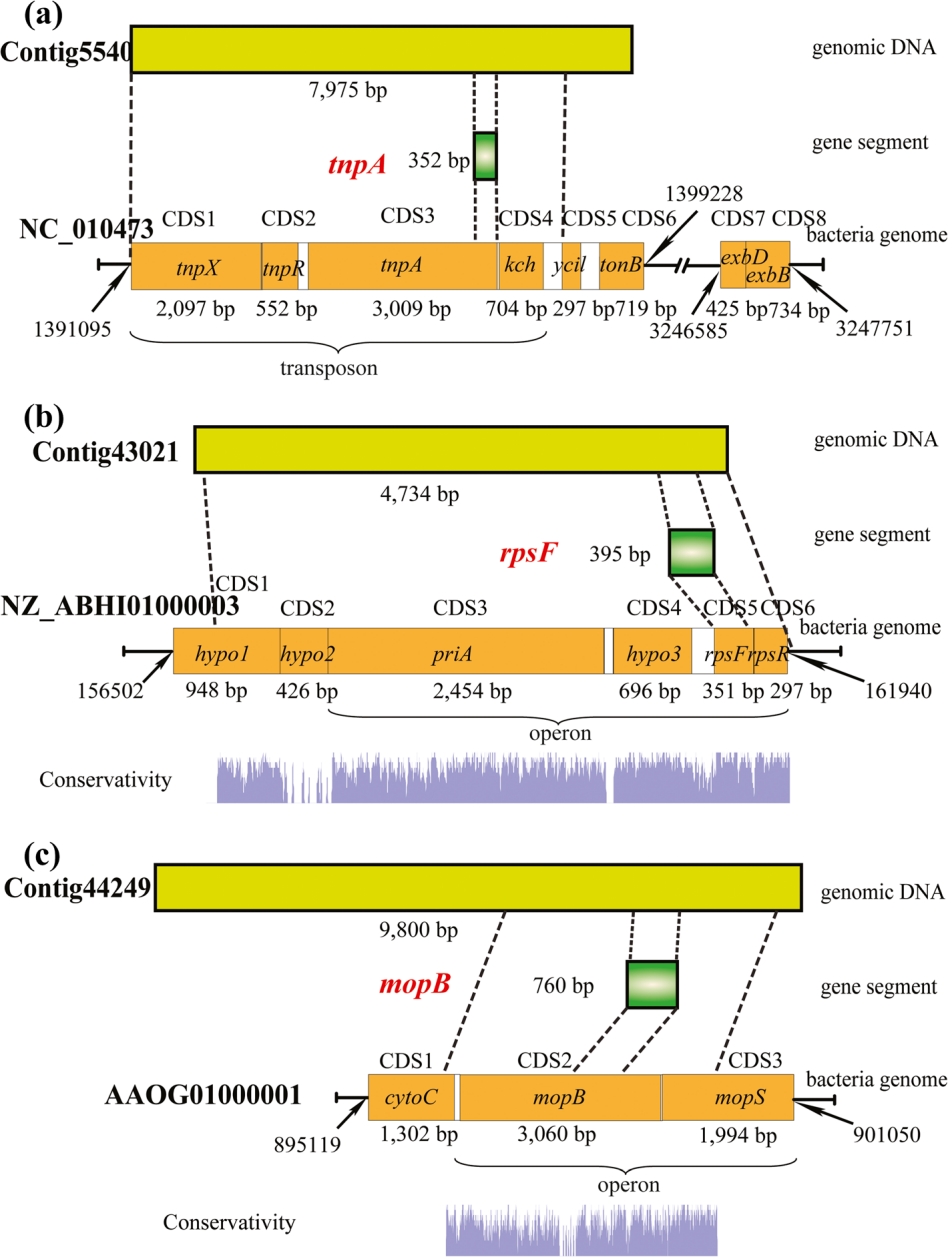
**Structures of three large horizontally transferred DNA fragments and their locations in both the donor and receptor genome.** The conserved regions between shrimp genomic contigs and corresponding donor genomes are displayed.

When comparing the donor genome with the shrimp genome contigs, three pairs of relatively large segments showed significant similarity around the position of HGT genes (Figure 3). HGT gene *tnpA*, which encodes a transposase, is derived from a transposon of *E. coli* that comprises three transposase-encoding genes (*tnpX*, *tnpR*, *tnpA*) and non-coding regions. In addition to the whole transposon, there was also a *kch* gene (encoding a voltage-gated potassium channel) downstream of the transposon, and the whole segment (6,913 bp) was completely integrated into the genome of *L. vannamei*. Transposons generally contribute little or nothing to the host’s phenotype, but some horizontally transferred transposons have been identified to benefit the recipient’s genomic evolution [30,31]. In addition to this large segment, there was one more gene, *tonB*, encoding a membrane spanning protein in the TonB-ExbB-ExbD complex [32], which was located downstream of the transposon in the donor genome. The other two genes (*exbB* and *exbD*, which encode ExbB and ExbD) were located far from *tonB* in the downstream. The *exbB* gene is one of the homologs of HGT gene *exbB* that was detected in this study (Table 1). It seems this horizontally transferred transposon might have served as a carrier for foreign genetic materials [31].

HGT gene *rpsF* was predicted to encode 30S ribosomal protein S6, which is involved in protein synthesis (Table 1). When compared with the most probable donor genome of *F. bacterium* (NZ_ABI01000003), it was found that *rpsF* was almost in full-length like that on the donor genome (Figure 3b). Furthermore, it was also completely mapped with shrimp genome contigs without any integrated introns. The corresponding regions of *rpsF* on the shrimp and bacterial genomes were highly conserved (E value of 2.00E-59 and identity of 87.5%), which indicates that *rpsF* is an intron-less gene that may have transferred from bacteria to *L. vannamei* recently. Around *rpsF*, there is a large segment on the shrimp genome that is similar to the donor genome (E-values ranged from 2.00E-75 to 1.00E-11, identity values ranged from 78.01% to 92.31%). This large segment, 4,655 bp in length, contains six genes on a corresponding donor bacterial genome (Figure 3). Besides, it is surprising to found that non-coding regions on this segment were not conserved, and also a predicted gene *hypo2* was not conserved as well. On donor bacterial genome, *hypo2* is overlapped with both upstream *hypo1* and downstream *prsN*. Homologous BLAST searching against the nt database detected a great many homologs (more than 100 matches) of this large segment, including *hypo2*. However, no evidence support *hypo2* is actually a coding gene. If it is a gene prediction error, *hypo2* may also a non-coding region. *F. bacterium* is just the best hit species of *rpsF* in GenBank database. It is known that there is a great deal of variation in genomes of bacteria. Therefore, the data of *F. bacterium* may be quite different from the true donor genome, which could lack *hypo2*. Thus, *hypo2* may be missed in donor genome before the transfer of this large segment into shrimp genome. Whatever, the alignment results indicated that all these non-coding regions were absent from shrimp genome contigs. Similar results were also observed around *mopB* (Figure 3c).

The third horizontally transferred large DNA segment (4,118 bp) was around HGT gene *mopB* (Figure 3c). *mopB* is predicted to encode molybdopterin oxidoreductase and iron-sulfur binding subunit. Another molybdopterin oxidoreductase encoding gene, *mopS*, is located downstream of *mopB* and is also transferred to the shrimp genome. There is a region of low conservation (267 bp) upstream of *mopB* between the shrimp genome contig and the most probable donor genome. The alignment results indicated that most of the sequence (79.4%) from this region was absent in the bacterial genome. Eukaryotic gene prediction software packages including AUGEST [33], Glimmer-HMM [34] and FGENESH [35] were used to predict exons in this shrimp genome contig, and all the prediction results indicated that the low conserved region was part of a complete exon but not an intron that had inserted into the gene. This suggested that shrimp might have redirected this gene to integrate an additional coding sequence into the gene.

### HGT genes under strong negative selection pressure

In order to identify the completeness of these HGT genes, we surveyed them from the genomic contigs with the help of gene prediction software packages AUGEST and FGENESH. Even though some of contigs are short in length, we still detect some HGT genes (*acsf*, *rpsF*, *exbB*, *mopB*, *rpsN*, *tnpA* and *dhfr*) have complete gene structure, including transcription start site, introns, extrons, polyA and stop codons. Moreover, comparing with the genes on the most probable donor genome, there are not any frameshifts among these HGT genes. Therefore, these HGT genes are relatively complete on shrimp genomes. Then, more efforts were made on the detection of whether HGT genes are exposed to selective pressure after the shrimps diverged from their ancestor. Through comparing HGT genes against the transcriptome unigenes of *Fenneropenaeus chinensis* and *Penaeus monodon* (two kinds of shrimps that phylogenetically close with *L. vannamei*), we collected 16 pairs of orthologous sequences (Table 2). Then, KaKs_Calculator [36] was used for calculating synonymous or nonsynonymous substitutions (d_S_, d_N_) among these pairs of genes. The statistical results showed that synonymous substitutions are relatively high in quantity (d_S_ is around 0.7). Except *stat*, almost all the ratios of the nonsynonymous substitutions per site to synonymous substitutions per site (d_N_/d_S_) among these pairs of HGT genes are significantly lower than 1 (Table 2), which indicated that most of these HGT genes are under strong negative selection (especially for *rpc2*, *sdrp*, *deha* and *omtp*, d_N_/d_S_ values lower than 0.1). Similar results have been found in HGT genes of aphid [37], the d_N_/d_S_ ratios of which are more than 0.3, which is larger than that of *L. vannamei*. Previous researches indicated that genes subjected to negative selection tend to maintain their functions [38]. Therefore, most of the proteins encoded by these negative selected HGT genes appear to be functional.

**Table 2:** **The d**_**N**_**/d**_**S **_**values of HGT genes**

		***F. chinensis***			***P. monodon***		
	**HGT genes**	**Alignment length**	**d**_**N**_**/d**_**S**_	**P value**	**Alignment length**	**d**_**N**_**/d**_**S**_	**P value**
	*acsf*	618	0.4694	5.49E-03	140	0.0010	2.76E-04
	*stat*	199	0.5162	2.22E-01	199	0.3882	8.54E-02
	*rpc2*	264	0.0527	1.54E-06	255	0.0516	6.28E-05
	*dhfr*	289	0.1057	3.42E-11	190	0.1123	2.77E-07
	*sdrp*	286	0.0752	5.22E-09	101	0.0488	8.54E-04
	*ankp*	384	0.4984	2.10E-04	148	0.3571	1.18E-01
	*deha*	574	0.0844	1.16E-17	317	0.1567	3.05E-05
***L. vannamei***	*omtp*	533	0.0850	5.60E-22	610	0.1263	1.13E-19

### Most HGT genes are predicted to be associated with energy metabolism and defense mechanism

**Figure 4:**
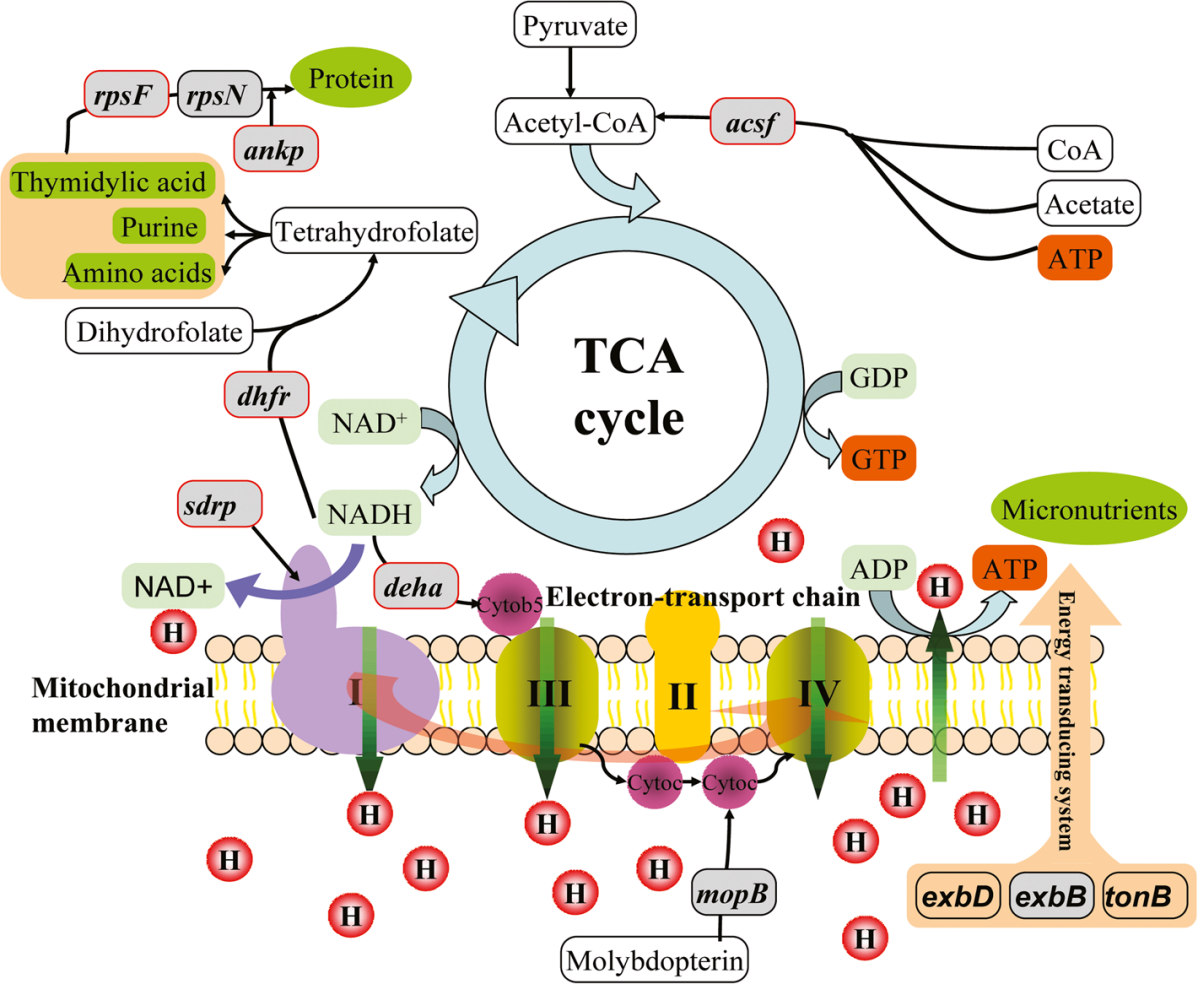
**Nine HGT genes participate in the TCA cycle and electron transport chain of the shrimp mitochondrion.** The majority of the functions of these HGT genes are predicted relate to energy metabolism. The HGT genes are shown in gray oblongs and the HGT genes differentially expressed during five development stages are marked with additional red borders.

Both TCA cycle and the electron-transport chain are predominant pathways of energy metabolism in the matrix of the mitochondria [39,40]. The HGT gene *acsf* is predicted to Acetyl-CoA synthetase, which is involved in the metabolism of carbohydrate during the first step of energy generation in the tricarboxylic acid cycle (TCA cycle) [41]. Other four HGT genes (*dhfr*, *sdrp*, *deha* and *mopB*) are predicted as genes in electron transport (Figure 4). *dhfr* is an HGT gene annotated to encode dihydrofolate reductase which can use NADH as an electron donor to produce tetrahydrofolate for certain amino acids synthesis in 1-carbon transfer chemistry [42], and *sdrp* are predicted to encode NAD(H) or NADP(H) oxidoreductases. *deha*, predicted to be an HGT gene from fungi, encodes a cytochrome b5 reductase-like enzyme that catalyzes the reduction of cytochrome b5 using NADH as the electron donor [43]. *mopB* is predicted to encode molybdopterin oxidoreductase which participates in transferring electrons to cytochrome c [44]. Furthermore, a gene encoding cytochrome c, which contains a heme-binding domain for energy production and conversion, is located upstream of *mopB* and was transferred along with *mopB* (Figure 3). And at last, the energy-transducing protein ExbB, encoded by HGT gene *exbB*, is a part of TonB-ExbB-ExbD complex to form an energy transduction system [45-47]. Therefore, according to the information of predicted functions, all these six HGT genes may associate with energy metabolism.

As for the six energy metabolism related HGT genes, it is of interest to find whether they are transferred from mitochondria in the distant past. It is well known that nuclear mitochondrial DNA (NUMTs) is commonly found to be transferred into nuclear genomes in various species [48]. However, in this study, none of six energy metabolism related HGT genes have been identified to show any similarity to the whole mitochondrial genome of *L. vannamei* (NCBI accession number of NC_009626.1). Therefore, unlike NUMTs, these HGT genes are not transferred from mitochondria in recent past, but transferred from non-mating organisms of shrimp in the long evolutionary history.

In Figure 4, the other three HGT genes are predicted to function in protein synthesis and interaction. *rpsF* and *rpsN* are predicted to encode two ribosomal protein subunits that are used for protein synthesis. *ankp* encodes a protein containing ankyrin repeats, which are used for mediating protein-protein interactions in diverse families of proteins [49]. Interestingly, the spread of ankyrin repeats has been suggested to have occurred by HGT, and their occurrence in yeast excludes exon shuffling [49]. This supported the HGT events of *ankp*, which maintained the stability of its gene structure.

According to the annotated functions of four of the HGT genes, they are associated with defense mechanisms. There are two antibiotic resistance genes (*stat* and *cata*) that are predicted to encode streptomycin 3′-adenylyltransferase and chloramphenicol acetyltransferase, respectively (Table 1). Both transferases are responsible for the streptomycin and chloramphenicol resistance and prevent them from binding to ribosomes [50,51]. Accidently, streptomycin and chloramphenicol are specifically bind to 30S and 50S ribosomal subunit, respectively, which are predicted encoded by two other HGT genes, *rpsF* and *rpsN* (Table 1). Thus *stat* and *cata* may be involved in protection of protein synthesis. As the only bacteria-to-Arthropoda HGT gene in *L. vannamei* (Figure 2), *omtp* was annotated to encode O-methyltransferase, which is ubiquitously dispersed in various organisms and plays an important role in animal growth, development and defense [52,53]. *rpc2* is annotated to encode repressor protein C2, which is a gene involved in the SOS response [54,55]. Generally, the SOS response is induced by various antibiotics, such as chloramphenicol, trimethoprim and streptomycin. The SOS response can promote HGT events of antibiotic resistance genes among bacteria [54]. Above all, the proteins encoded by these four HGT genes are predicted as defense-related.

### Gene expression of the HGT genes in differential developmental stages of shrimp

**Figure 5:**
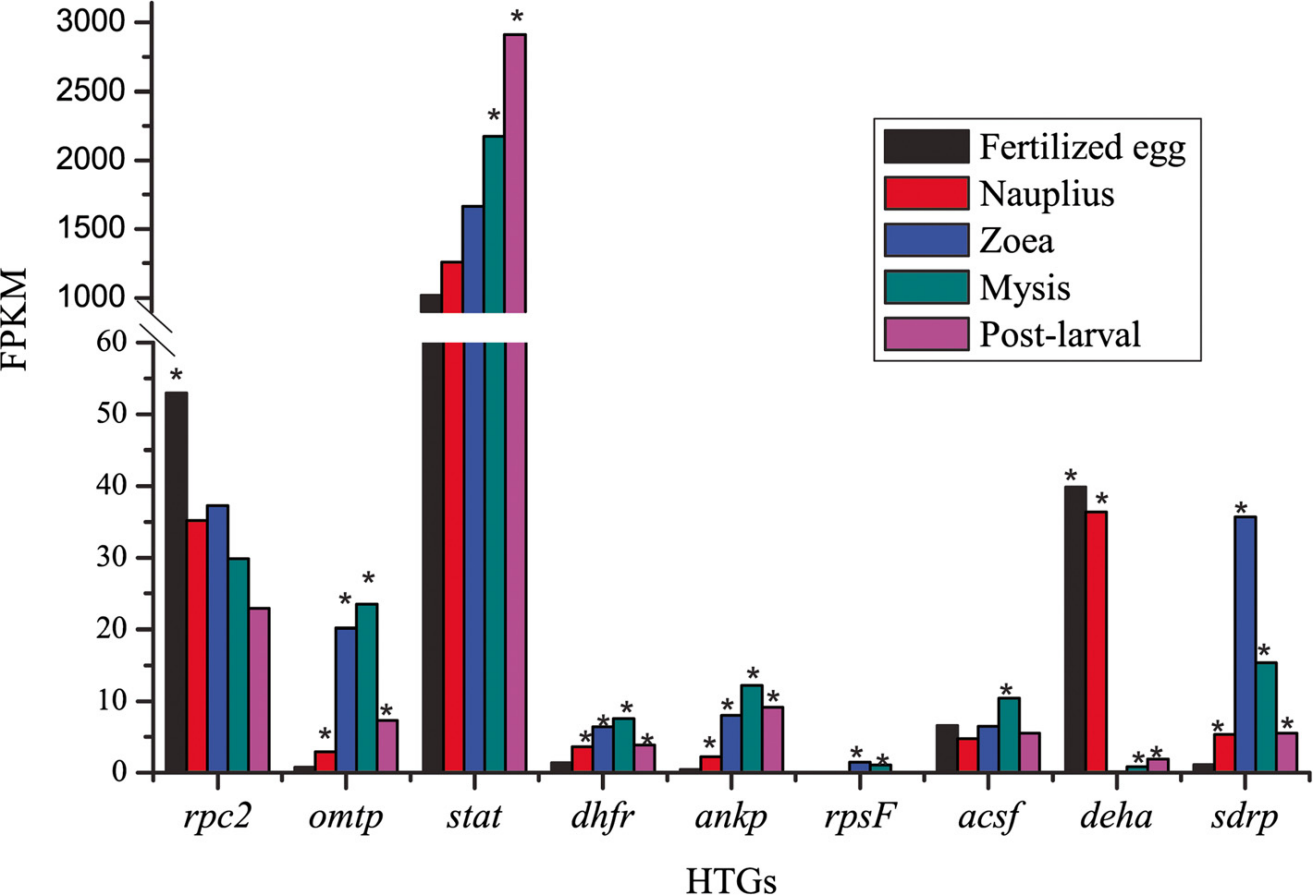
**Differential expression of eight HGT genes at five developmental stages of shrimp.** Gene expression level was evaluated by FPKM value. * indicates the HGT genes were significantly differentially expressed when compared with the lowest expressed development stage.

To detect whether HGT genes have effects on shrimp growth and development, we performed differential gene expression (DGE) analysis on these HGT genes, based on the transcriptomes of five developmental stages of shrimp larvae (see Methods). The number of reads aligned to the transcripts were calculated (Additional file 1: Table S4), and the expression level of each transcript was measured by FPKM values (fragments per kilobase of exon per million fragments mapped). High coverage of reads successfully supported for the following DGE analysis. Nine HGT genes showed differential expression among five stages (Figure 5, Additional file 1: Table S5). *stat* displayed the highest expression level (FPKM values ranged from 1018.57 to 2910.86) at all five stages, while *rpsF* was expressed at the lowest (FPKM values ranged from 0 to 1.43). *rpsF* seems only expressed at the stage of zoea and mysis. Similar to *rpsF*, the other two fundamental DNA and protein synthesis related HGT genes (*dhfr* and *ankp*) displayed an expression pattern that gene expression level at the stage of zoea and mysis was generously higher than the other stages (Figure 5, Additional file 1: Table S4). In addition to providing fundamental nucleic acid precursors and certain amino acids, *dhfr* also participates in energy metabolism. Three other energy metabolism-related HGT genes showed DGE, and two of them (*acsf* and *sdrp*) displayed similar expression pattern with *dhfr*. *deha* showed the reverse expression pattern, in that it showed lower expression at stages of zoea and mysis than at other stages (Figure 5). In addition, three defense-related HGT genes (*rpc2*, *omtp* and *stat*) were universally more highly expressed than other HGT genes (Figure 5, Additional file 1: Table S5), indicating that they may play an important role in early shrimp development.

## Discussion

### Structure alteration of HGT genes indicated the mechanisms of HGT

Previous research on HGT events suggested that some of the HGT genes transferred from bacteria acquired eukaryotic features, such as signal peptides and spliceosomal-type introns [13,37]. Similar occurrences were observed in the present work: five spliceosomal-type introns are integrated into four HGT genes in the shrimp genome. Two HGT genes originating from fungi (*ankp* and *deha*) seem to have differential introns to that of the most probable donor, while the other two HGT genes (*acsf* and *sdrp*) originated from bacteria and new introns were directly inserted into the HGT genes. As for *ankp* and *deha*, the introns on it may exchanged between donor and recipient genome, otherwise, it may originated from an un-sequenced fungus, of which the intron regions are also similar to that of shrimp genome. Whereas, the introns on *ankp* and *deha* are predicted to be highly repeated in shrimp genome, which indicated these introns maybe arose from the shrimp. Spliceosomal introns in eukaryotic genes have been identified as a possible barrier to HGT from fungi. However, it would still possible for fungi-originated genes transfer through mediation of spliced RNA intermediate [1]. Thus, the introns on the two fungi-originated genes (*ankp* and *deha*) are most probably exchanged with shrimp introns rather than originated from un-sequenced fungi. Although introns are generally thought to be mildly deleterious elements for mature mRNA generation, there are still indications that some introns are essential for eukaryote genomes, e.g. alternative splicing, RNA editing and enhancing mRNA transcription [56]. Thus, the introns integrated into these HGT genes may play essential roles in gene expression and regulation. In addition to introns, other non-coding DNA and even coding regions were changed in the HGT genes. The horizontally transferred large segment around *rpsF* was observed to exclude non-coding regions in the shrimp genome (Figure 3). By contrast, an inserted coding region was detected at the 5′ end of *mopB* in the shrimp genome. Although there is no evidence that this inserted coding sequence enhances the protein expression level or function, it may undergo an adaptive fitness in shrimp [57].

There are several mechanisms to explain HGT events in eukaryotes. The mechanism of bacteria-to-animal HGT most probably arises from endosymbiosis [1,58]. The close and constant proximity of cells provides a platform for the transfer of genetic materials. Generally, the genetic materials that horizontally transferred are mostly complete or fragments of genes, but not large DNA fragments. However, horizontally transferred large DNA fragments were detected in previous studies and in the present study [59-62]. In the case of single HGT genes, spliceosomal-type introns were found, which indicated that these genes acquired the introns for their function after they were transferred to the host genome. In a previous study [63], several conserved introns in both recipient and donor taxa were found in HGT genes, which indicated that those HGT genes are the products of direct eukaryote-to-eukaryote transfer. By contrast, in this study, the introns in two of the fungi-to-*L. vannamei* HGT genes seem to be exchanged, which suggested the introns may have not have transferred into host genomes along with the exons. The single HGT genes, including intron-free HGT genes, were most probably acquired from mature RNA after gene transcription in the donor cell. It is also possible that these genes were acquired from raw DNA segments and shrimp could recognize the splice sites accurately and substitute with its own introns, which seems mechanistically unlikely. Operons have been reported playing important role in transfer of large DNA segments which include three or more genes [64]. Through operon prediction by DOOR (Database of prOkaryotic OpeRons) [65], we found that this large horizontally transferred segment around *rpsF* was predicted as an operon. Furthermore, there is a gene *priA*, located upstream of this operon, encoded primosomal protein that are responsible for creating RNA primers during DNA replication [66]. Primosomal proteins are essential for operon HGT events, and actually, primosomal operon have ever been detected in previous researches [67]. Therefore, it is reasonable to think that the large segment around *rpsF* is completely transferred. As for the large horizontally transferred segment around *mopB*, operon prediction indicated that *mopB* and *mopS* are also involved in an operon, which implies that they are horizontally transferred together (Figure 3c). Although these large segments are transferred together, non-coding regions were removed from them (Figure 3, the large segment around *tnpA* was an exception because it was transferred as a single transposon), which suggested intergenic regions may not transferred into the shrimp genome. This supports the hypothesis that mature RNA was the main substrate for HGT.

### Some HGT genes appeared to be functional in *L. vannamei*

Given that shrimp cells are frequently in proximity to prokaryotic cells, it is not surprising that HGT can be detected between them. The type of prokaryote-to-eukaryote HGT is generally of interest because it could potentially providing novel functions to eukaryotes, allow adaptation to novel niches, and affect their evolution [1]. However, finding prokaryotic DNA in eukaryotic genome is not sufficient to imply that this DNA is functional in the host genome. In this study, several evidences suggest the functionality of these HGT genes for shrimp. Firstly, most of HGT genes had complete gene structure, and none of frameshift mutations could be found among them, which indicated they are not pseudogenes. Secondly, selection pressure analysis of these HGT genes showed that there was a strong negative selection for most of them (d_N_/d_S_ values significantly lower than 1), indicating deleterious effects for most mutations at the protein levels [38], thus, these HGT genes in shrimp seemed to be effectively functional after the transfer. Lastly, these HGT sequences were derived from EST or transcriptome sequences, which imply they could transcribe in shrimp cells. Furthermore, most of the HGT genes were found to be transcribed at the early development stages of shrimp larvae, and they displayed significant DGE at five development stages. This indicated that these HGT genes were tightly associated with the development of shrimp. Therefore, these genes transferred from bacteria or fungi appeared to be functional in shrimp cells. According to the predicted functions of these HGT genes, six of them were annotated as energy metabolism related, and four of them were predicted to be associated with defense of shrimp. Among them, one of defense-related HGT gene, *omtp*, was reported to be involved in defense response against bacterial infection in Chinese shrimp, *F. chinensis*[28]. In a previous report [68], there was strong evidence to show that invertebrates could obtain defensive complex polyketides from bacterial symbionts via O-methyltransferase (encoded by *omtp*) methylation of the marine compounds. Therefore, *omtp* was assumed to be an effective functional defense gene transferred from bacteria. Given the unlikely transfer and transcription of genes that in the donor are related to energy metabolism and defense, we speculate that these HGT genes may have the same role in the recipient. This is consistent with what is known about the demands of shrimp where molting is energy intensive and current aquaculture methods stressful [14,16,69]. However, the precise roles of these proteins need to be demonstrated through further studies.

## Conclusions

Through sequence homology comparison, phylogenetic analysis and experimental verification, fourteen bacteria or fungi originated HGT genes were detected in *L. vannamei*. Spliceosomal-type introns were found to be inserted in four HGT genes, while non-coding regions of two large horizontally transferred segments were lacked on the shrimp genome. These structure alterations provide evidence that mature RNA may be the substrate for HGT events. Among 14 HGT genes, most of them were detected to be exposed to negative selection pressure and they appeared to be functional. Functions prediction annotated them to be associated with energy metabolism and defense of shrimp. Further studies should be taken to demonstrate the precise roles of these HGT genes.

## Methods

### Sources of genome and transcriptome dataset of *L. vannamei*

A total of 1.9 Gb shrimp genome contigs, which cover approximately 76% of the whole genome, were assembled from 42-fold coverage of whole genome shotgun reads of *L. vannamei* in our laboratory. The genome contigs were used for mapping of the candidate HGT genes on the shrimp genome. Information on the genome contigs to which candidate HGT genes mapped were submitted to NCBI (http://www.ncbi.nlm.nih.gov, KC701594 - KC701607).

The gene set of *L. vannamei* was predominantly composed of two online datasets from NCBI: 161,796 ESTs that were generated from multiple tissues [20,70], and 74,111 transcriptome sequences that were generated from the whole body of *L. vannamei* larvae [21]. The ESTs were assembled into 32,550 unigenes using CAP3 with default parameters [71]. The software Minimus2 (http://sourceforge.net/apps/mediawiki/amos/index.php?title=Minimus2) was then used to merging the two sequence sets and remove duplicated, redundant sequences. Ultimately, 82,579 gene segments were generated and used for subsequent HGT genes detection.

### Local protein database construction

There are 6,529,500 protein sequences collected from the complete proteomes of 4,080 species on the NCBI ftp site (ftp.ncbi.nlm.nih.gov), and these were used for HGT detection. The constructed local database included seven arthropods, 32 fungi, seven plants, 31 other eukaryotes, 1,607 bacteria and 2,387 viruses (Additional file 1: Table S1).

### BLASTx-based HGT search and detection

BLASTx-based [72] homologous sequence searching was the first procedure for HGT detection, and there were three steps of similar sequences identification in the detection pipeline (Figure 1). First, each candidate *L. vannamei* gene segment was compared with the total protein sequences in the local database with cutoff thresholds of E value ≤ 1E-20, identity value ≥ 25% and overlap value ≥ 25 [72]. The remaining 13,388 highest similarity sequences were extracted for further analysis. The second step was the similarity comparison between arthropods and other species. By comparing the BLAST results, candidate genes with higher BLASTx similarity scores to Arthropoda than other species were excluded because these genes were most probably the Arthropoda unique genes. In step three, using the same cutoff thresholds as the first step, the remaining sequences were used to search against the nr database. If there were less than 10 homologs in one phylogeny, the corresponding gene segments were not considered for subsequent phylogenetic analysis.

### Phylogenetic analysis

Homologous sequences of the remaining candidate HGT genes were extracted from the nr database, and the candidate HGT genes were translated from nucleotides to amino acid sequences. The combination of the candidate HGT genes sequences and their homologs from the nr database were used to construct phylogenetic trees, which were used to assess standard HGT or non-HGT events [22,23]. Three types of phylogenetic tree were constructed based on different methods and were compared with each other to generate the best phylogenetic topology.

The first type of tree was generated by complement alignment of the sequences using ClustalX [73], and computing the pairwise distance-matrix of the aligned sequences with PHYLIP software package [74]. A neighbor-joining tree was then constructed with the distance-matrix and 1000 bootstrap replicates were performed. The alignment method of the other two phylogenetic analyses was implemented on MUSCLE 3.6 [75] and the conserved region of each alignment was trimmed using Gblocks [76], which allows less strict flanking positions and gap positions within the final blocks, but does not allow many contiguous nonconserved positions. Using Phyml [77], we performed an approximate likelihood-ratio test (aLRT, Minimum of SH-like and Chi2-based parametric) on the conserved aligned sequences [78], and constructed the phylogenetic tree with the substitution model of JTT. The maximum likelihood (ML) phylogeny was the last type of analysis, and the ML trees are shown in the figures. ML trees were constructed by Phyml using a WAG + gamma +Inv model, and 100% bootstraps were performed to gain the branch support values [8,22]. The phylogenetic trees were used to judge HGT events and HGT directions, based on previous studies that considered that HGT genes tend to form a monophyletic branch with a set of far-related species, which are considered as the donors [22,23]. The alignment datasets and phylogenetic tree-files of the HGT genes from analyses have been deposited in TreeBASE as study S14162 (http://www.treebase.org/treebase/). To test the support for contentious topology of the extracted HGT genes, Bayesian phylogenetic inference (BI) was performed on them with the help of the program Mrbayes 3.2.1 [79]. In the BI analysis, two independent runs, each with four chains, were analyzed for millions of generations until the standard deviation of split frequencies converged towards zero. A burn-in of 25% samples is used for summarizing the parameter values and trees.

### Verification of HGT genes by PCR

ESTs and transcriptome sequences may be contaminated by other organisms in the procedures of RNA extraction, library construction and sequencing; therefore, the candidate HGT genes were verified to confirm whether they were part of the genome of *L. vannamei*. Each candidate sequence that showed significant HGT events was compared against the draft genome contigs using BLASTn with a cutoff threshold of E value ≤ 1E-40, identity value ≥ 90% [72]. To avoid the effects of genome mis-assembly, a DNA-based PCR was implemented to amplify the genomic segments containing HGT genes. DNA was isolated from the muscle tissue of the adult shrimps. The information of the genome contigs including HGT genes were extracted for primer design and the information of the primer pairs for each HGT gene are listed in Additional file 1: Table S2. Besides, PCR amplification was also performed on the edge of HGT fragments on genome contigs for the attempts of amplify both HGT region and eukaryotic portion of the genome. The edges of the HGT fragments were determined based on the BLAST (E value ≤ 1E-5) of the genome contigs against the most probable donor genome. The edges were the initial sites of two sides which showed homologous with the most probable donor genome. The primers were designed to amplify the products covering both HGT and eukaryotic portions as long as possible on both sides of the HGT region. The edge information and the primer pairs are listed in Additional file 1: Table S3. The PCR parameters were 94°C for 3 min; 35 cycles of 94°C for 30 s, annealing temperature (It is different for each product and are listed in Table S2-S3) for 30 s, and 72°C for 45 s; and a final 72°C for 7 min. The PCRs were conducted using ABI GeneAmp PCR System 9700 (Applied Biosystems, Foster City, CA, USA). Besides, negative controls were set using *Escherichia coli* genomic DNA or distilled water as template for PCR amplification, and a positive control were performed using primers designed in previous study, which has been successfully amplified a specific shrimp genomic sequence (GI:124633758) [80]. All the PCR products were sequenced to test their integrity and accuracy using ABI 3730xl DNA Analyzer (Applied Biosystems, Foster City, CA, USA). The sequences generated from sequencer were compared with the original genomic contigs.

### Structure analysis of HGT genes

The structures of HGT segments and their surrounding sequences on both the shrimp and donor genome were analyzed. The tBLASTx program was used to compare the HGT segments and shrimp genome contigs against the complete donor genomes with an E value cutoff of 1.00E-10 in consideration of a longer match length. The up- and downstream genes of the HGT genes positions on the donor genome were identified and analyzed to determine whether they were co-transferred to shrimp genome. Using ClustalX, the conservation between shrimp genome contigs and donor genomes was displayed along the alignment sequences.

### Calculation of d_N_/d_S_ ratios

We used KaKs_Calculator to calculate synonymous and nonsynonymous substitutions between orthologous pairs to identify whether there is natural selection subjected to these genes [36]. The 46,676 transcriptome unigenes of *F. chinensis*[81] and 7.8 Gb transcriptome reads of *P. monodon* were downloaded from NCBI (SRX110652, SRX110651 and SRX110649). The transcriptome reads of *P. monodon* were assembled into 182,648 unigenes through using the Trinity program [82]. BLASTn-based homologous sequence searching was performed for predicted HGT genes of *L. vannamei* against the transcriptome unigenes of *F. chinensis* and *P. monodon*. The homologous sequences of each HGT gene were aligned using ClustalX and the open reading frames were determined, then, we calculated the d_N_/d_S_ for each orthologous pair using YN algorithm with KaKs_Calculator.

### DGE profiles of HGT genes

Recently, five transcriptomes of *L. vannamei* were sequenced at different developmental stages in our laboratory: fertilized egg; nauplius; zoea; mysis and post-larval [16]. More than 23.7 Gb of high quality clean data were generated and each library contained a mean number of 52,660,075 reads. All the reads were then assembled into 66,815 unigenes using the Trinity program [82]. The HGT related transcriptome reads have been submitted to NCBI SRA database with the accession number of SRR839222, SRR839236, SRR842572, SRR842625 and SRR842627. The ESTs, transcriptome sequences of *L. vannamei* on NCBI, and our transcriptome sequences were generated from different individuals; therefore, the DGE analysis will be affected when analyzing the HGT gene segments directly. Therefore, the subsequent DGE analysis of HGT genes was represented by that of their extremely homologous unigenes in five transcriptomes. For each transcriptome, all reads were processed and aligned to all unigenes using SOAPaligner v2.21 which allow two base mismatches, and coverage of every unigene was analyzed. The transcript abundance was measured by FPKM values:

$$\mathrm{FPKM}=\frac{10^{6}C}{\mathrm{NL}/10^{3}}$$

Set FPKM to be the expression of unigene A, and C to be number of fragments that uniquely aligned to unigene A, N to be total number of fragments that uniquely aligned to all unigenes, and L to be the base number in the CDS of unigene A. The FPKM method is able to eliminate the influence of different gene length and sequencing level on the calculation of gene expression. As the data came from five developmental stages for DGE analysis, with the help of the edgeR package [83], we performed 10 pairwise comparisons of the expression between any two developmental stages of shrimp. The DGE ratios, which were calculated using the ratio of the normalized FPKM values from any two samples, were used to test the statistical significance, as described recently [84,85]. The p-values were analyzed using the edgeR package for significant differences, and q-values were calculated by applying a false discovery rate (FDR) adjustment for multiple testing [86,87]. The criteria for DGE evaluation are DGE ratio ≥ 2, p value ≤ 0.001 and FDR ≤ 0.001.

## Abbreviations

HGT: Horizontal gene transfer; ESTs: Expression sequence tags; DGE: Differential gene expression.

## Competing interests

The authors have declared that no competing interest exists.

## Authors’ contributions

JHX designed the study. JBY carried out the analysis, drafted and revised the manuscript. XJZ revised the manuscript. JKW carried out partial analysis. XJZ, CZL, FHL and JHX supervised the study. All authors read and approved the final manuscript.

## Supplementary Material

Additional file 1: Table S1 Local genome database for HGT identification. **Table S2**. Primers of the 14 HGT genes for PCR. **Table S3**. The PCR primers of the edge products of 14 HGT genes. **Table S4**. The FPKMs of each HGT genes in different development stage. **Table S5**. Differential expression of the 14 HGT genes at five development stages of shrimp larvae. Click here for file

Additional file 2: Figure S1 Phylogenetic tree of *acsf* and its homologs. **Figure S2**. Phylogenetic tree of *rpsF* and its homologs. **Figure S3**. Phylogenetic tree of *rpsN* and its homologs. **Figure S4**. Phylogenetic tree of *exbB* and its homologs. **Figure S5**. Phylogenetic tree of *mopB* and its homologs. **Figure S6**. Phylogenetic tree of *tnpA* and its homologs. **Figure S7**. Phylogenetic tree of *stat* and its homologs. **Figure S8**. Phylogenetic tree of *rpc2* and its homologs. **Figure S9**. Phylogenetic tree of *dhfr* and its homologs. **Figure S10**. Phylogenetic tree of *cata* and its homologs. **Figure S11**. Phylogenetic tree of *sdrp* and its homologs. **Figure S12**. Phylogenetic tree of *ankp* and its homologs. **Figure S13**. Phylogenetic tree of *deha* and its homologs. Click here for file
