# Supplementary material for: Horizontally transferred genes in the genome of Pacific white shrimp, Litopenaeus vannamei
Source: BMC Evol Biol. 2013 Aug 6;13:165. doi: 10.1186/1471-2148-13-165 (PMC3750580; doi:10.1186/1471-2148-13-165)
Supplement: Additional file 2: Figure S1 — Phylogenetic tree of acsf and its homologs. Figure S2. Phylogenetic tree of rpsF and its homologs. Figure S3. Phylogenetic tree of rpsN and its homologs. Figure S4. Phylogenetic tree of exbB and its homologs. Figure S5. Phylogenetic tree of mopB and its homologs. Figure S6. Phylogenetic tree of tnpA and its homologs. Figure S7. Phylogenetic tree of stat and its homologs. Figure S8. Phylogenetic tree of rpc2 and its homologs. Figure S9. Phylogenetic tree of dhfr and its homologs. Figure S10. Phylogenetic tree of cata and its homologs. Figure S11. Phylogenetic tree of sdrp and its homologs. Figure S12. Phylogenetic tree of ankp and its homologs. Figure S13. Phylogenetic tree of deha and its homologs. [file 1471-2148-13-165-S2.doc]

**
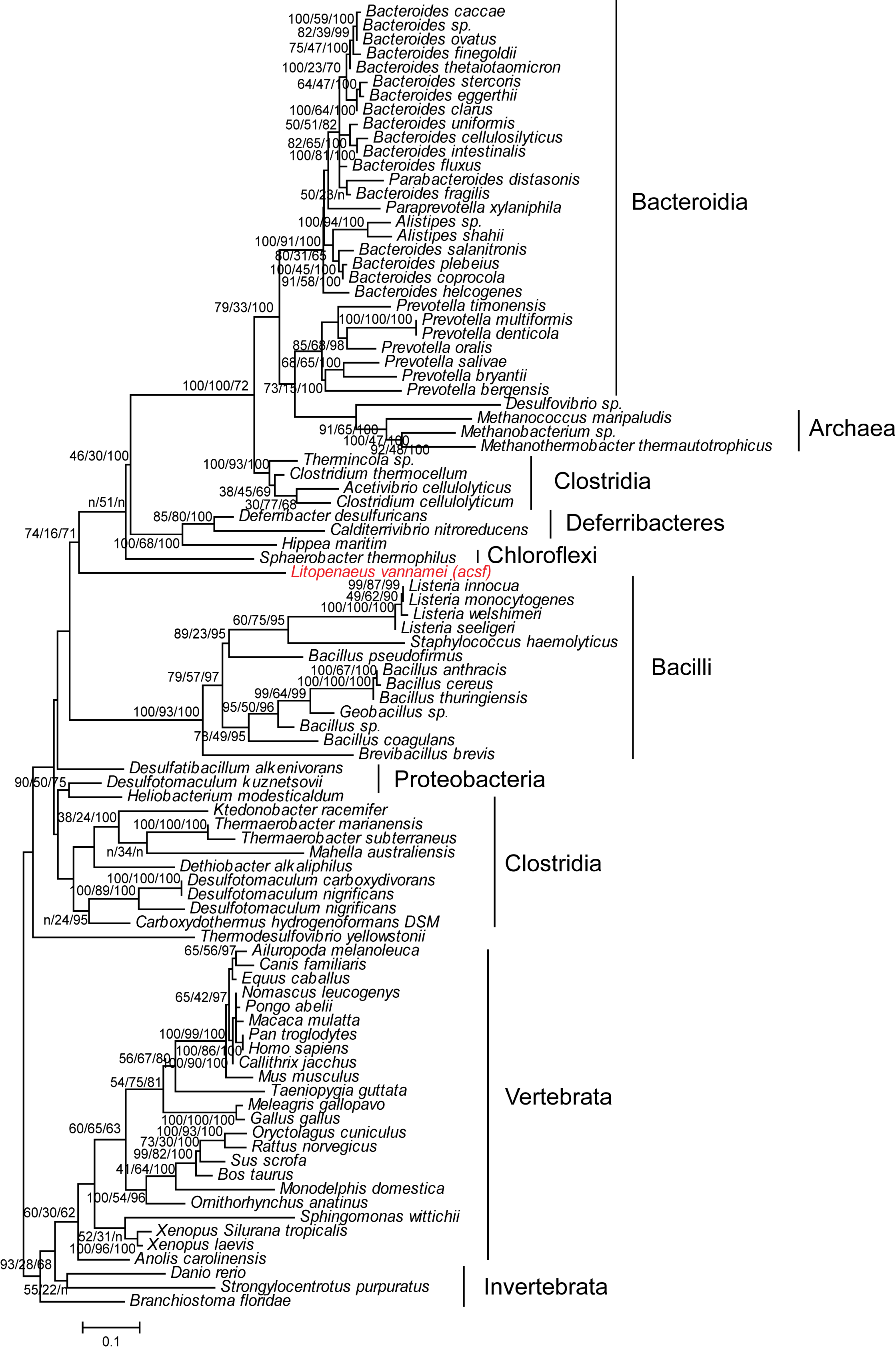
**

Figure S1. Phylogenetic tree of *acsf* and its homologs. The support values of ML, NJ and BI analysis displayed beside each node.

**
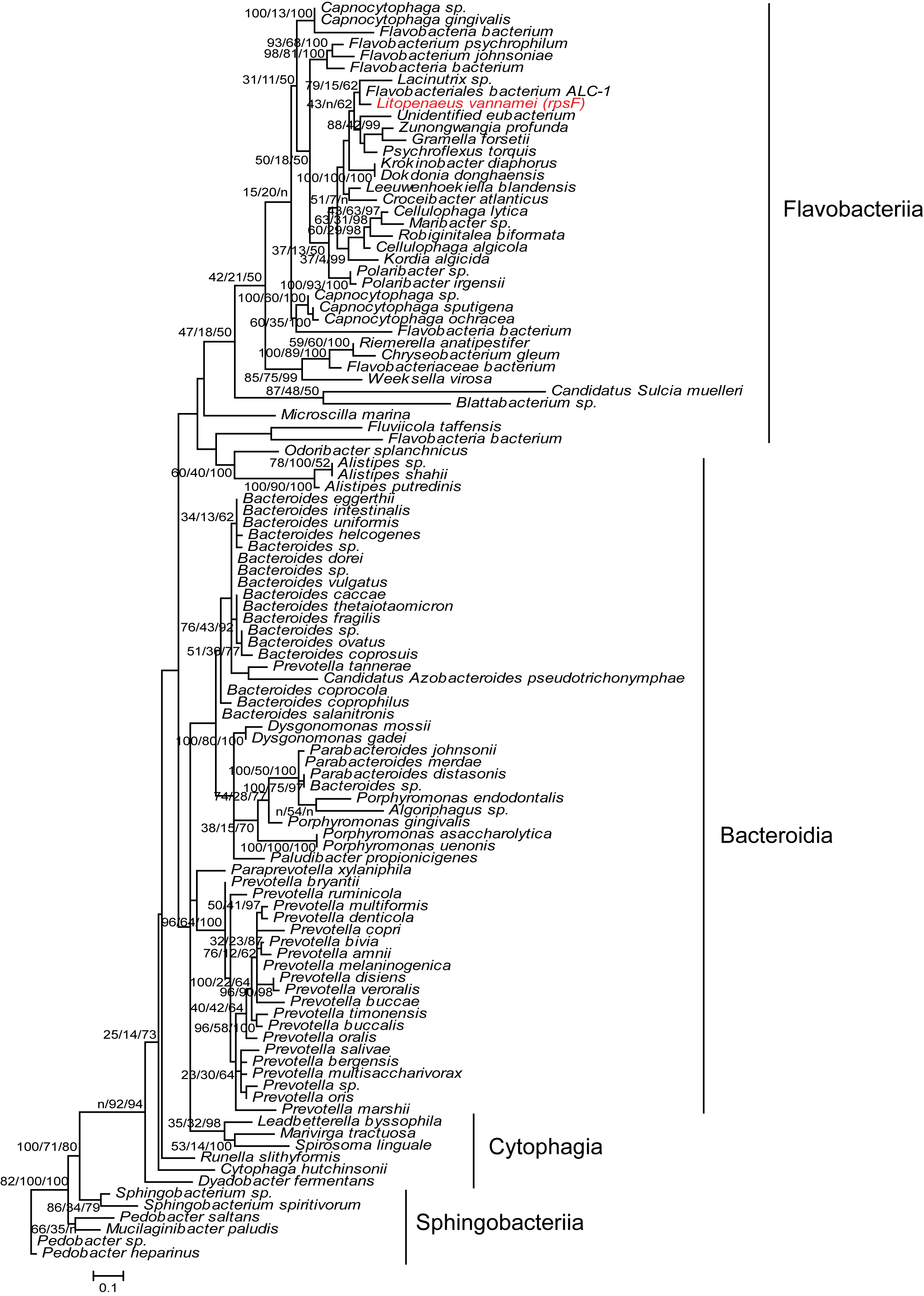
**

Figure S2. Phylogenetic tree of *rpsF* and its homologs. The support values of ML, NJ and BI analysis displayed beside each node.

**
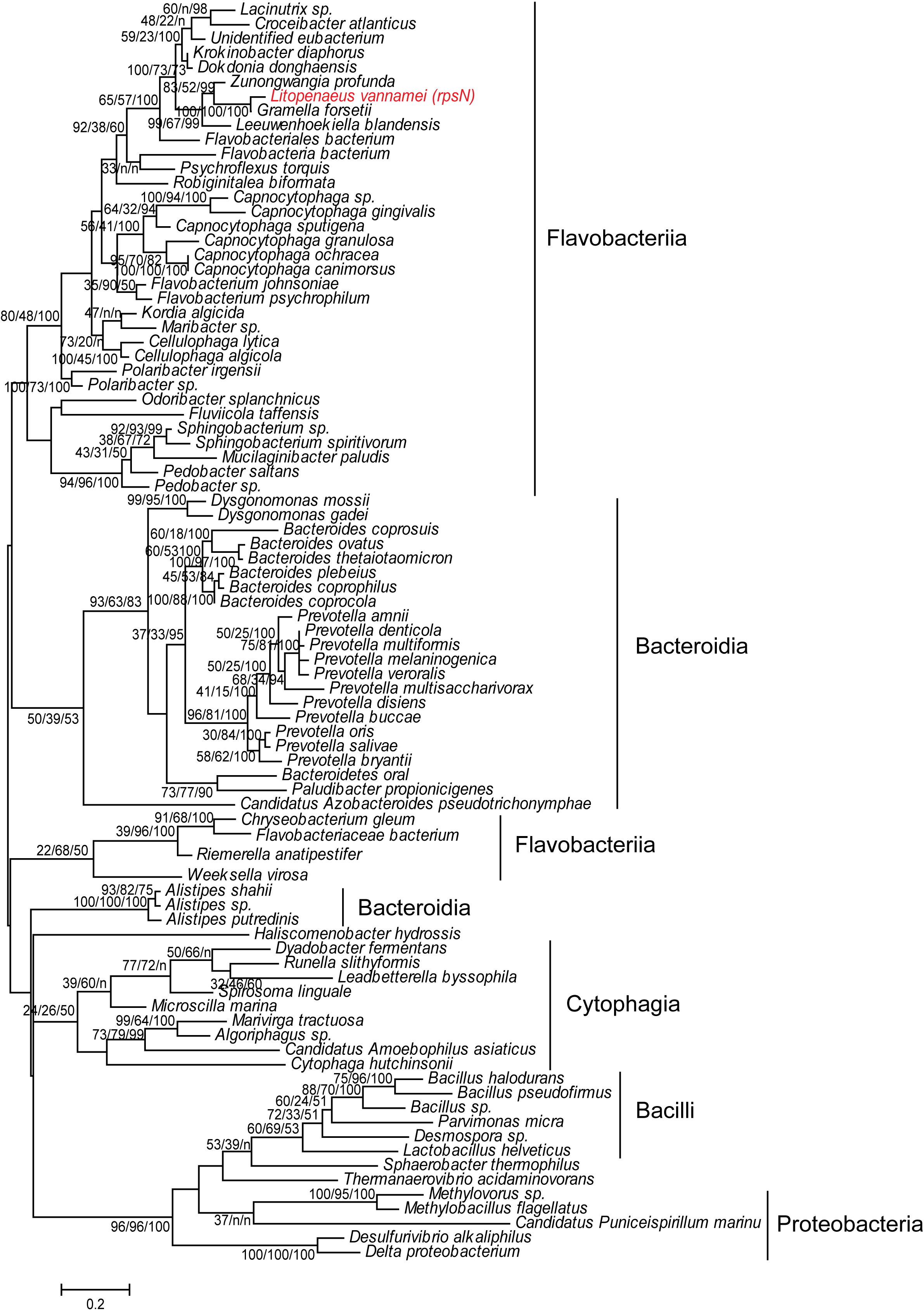
**

Figure S3. Phylogenetic tree of *rpsN* and its homologs. The support values of ML, NJ and BI analysis displayed beside each node.

**
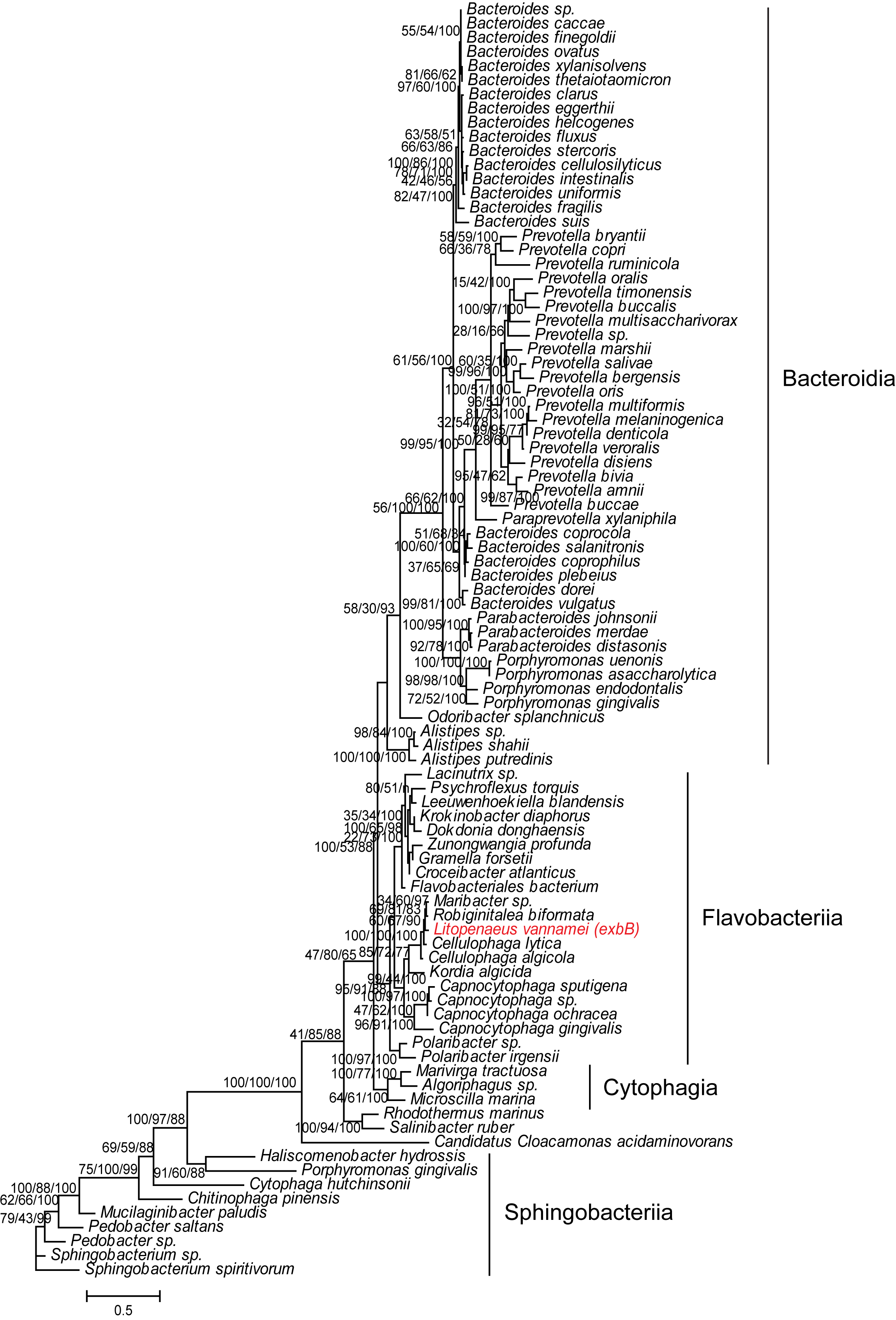
**

Figure S4. Phylogenetic tree of *exbB* and its homologs. The support values of ML, NJ and BI analysis displayed beside each node.

**
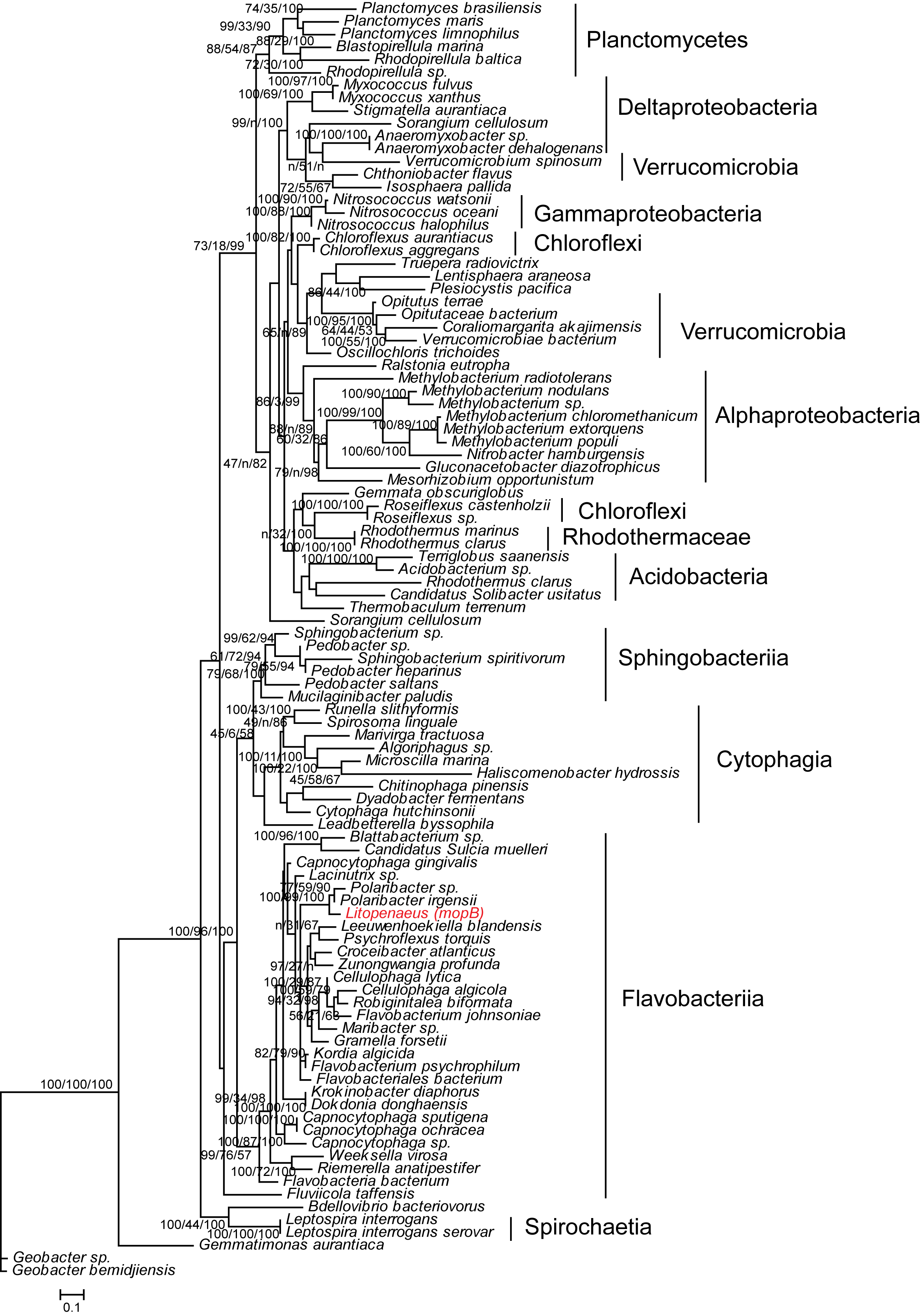
**

Figure S5. Phylogenetic tree of *mopB* and its homologs. The support values of ML, NJ and BI analysis displayed beside each node.

**
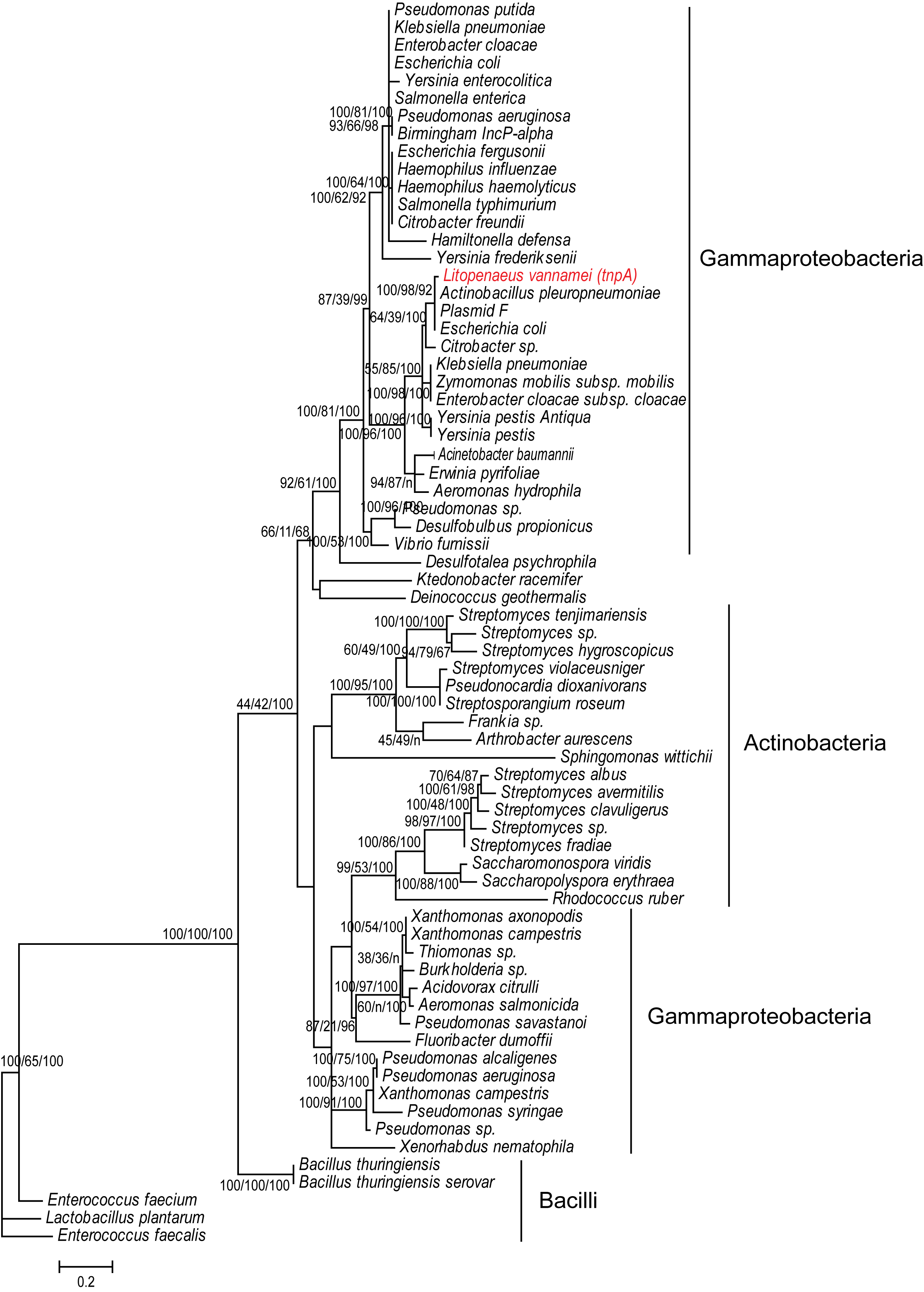
**

Figure S6. Phylogenetic tree of *tnpA* and its homologs. The support values of ML, NJ and BI analysis displayed beside each node.

**
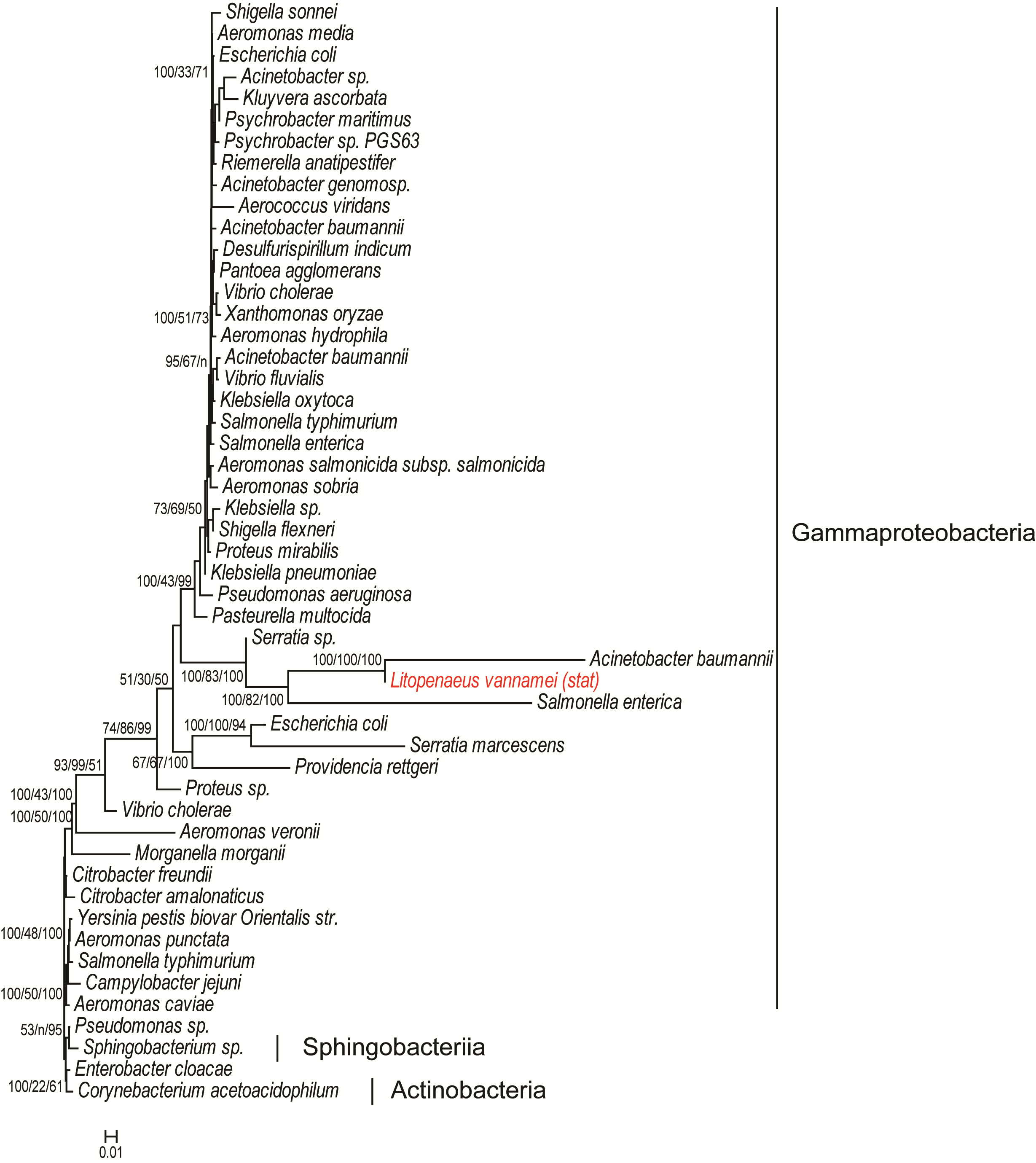
**

Figure S7. Phylogenetic tree of *stat* and its homologs. The support values of ML, NJ and BI analysis displayed beside each node.

**
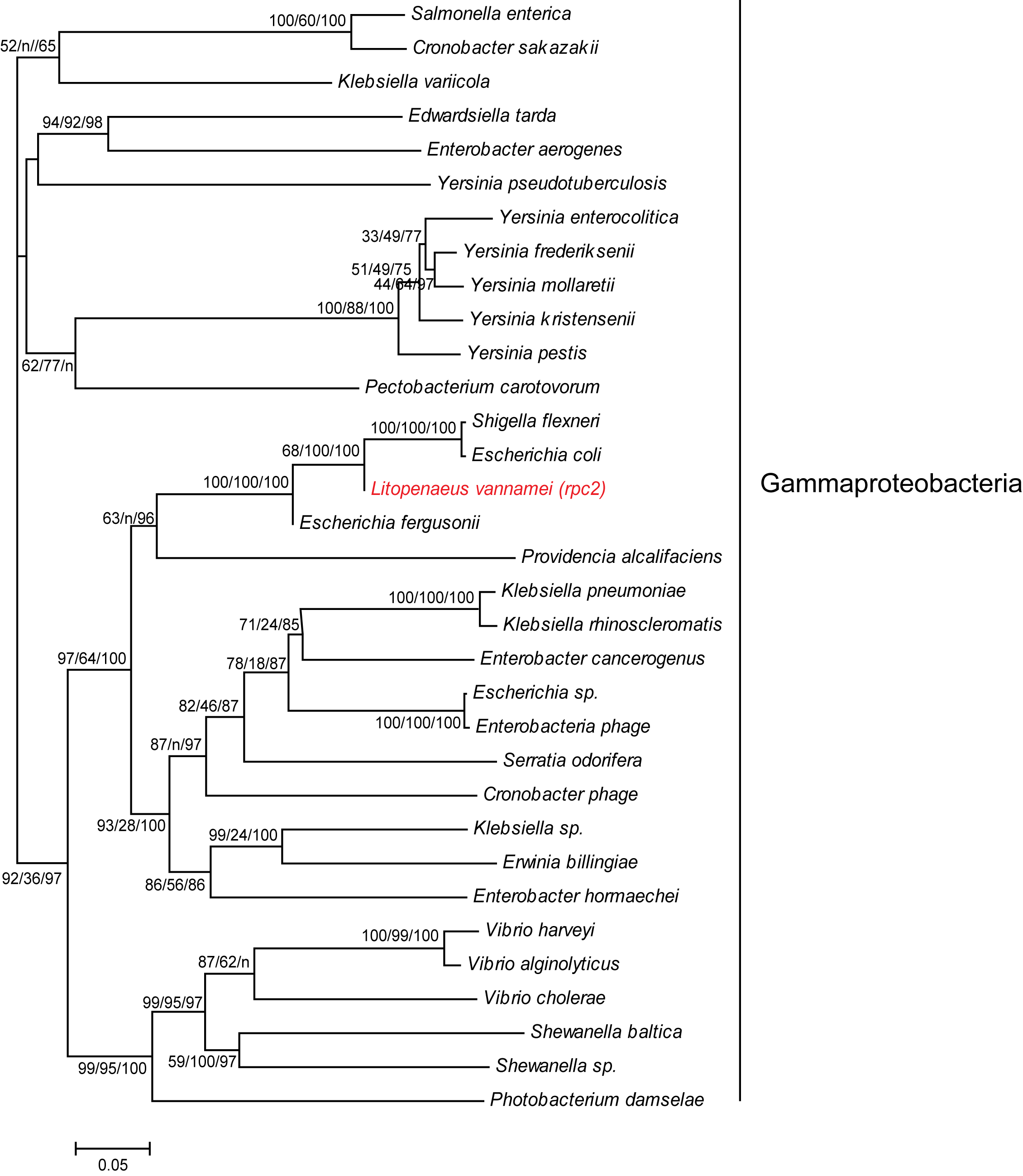
**

Figure S8. Phylogenetic tree of *rpc2* and its homologs. The support values of ML, NJ and BI analysis displayed beside each node.

**
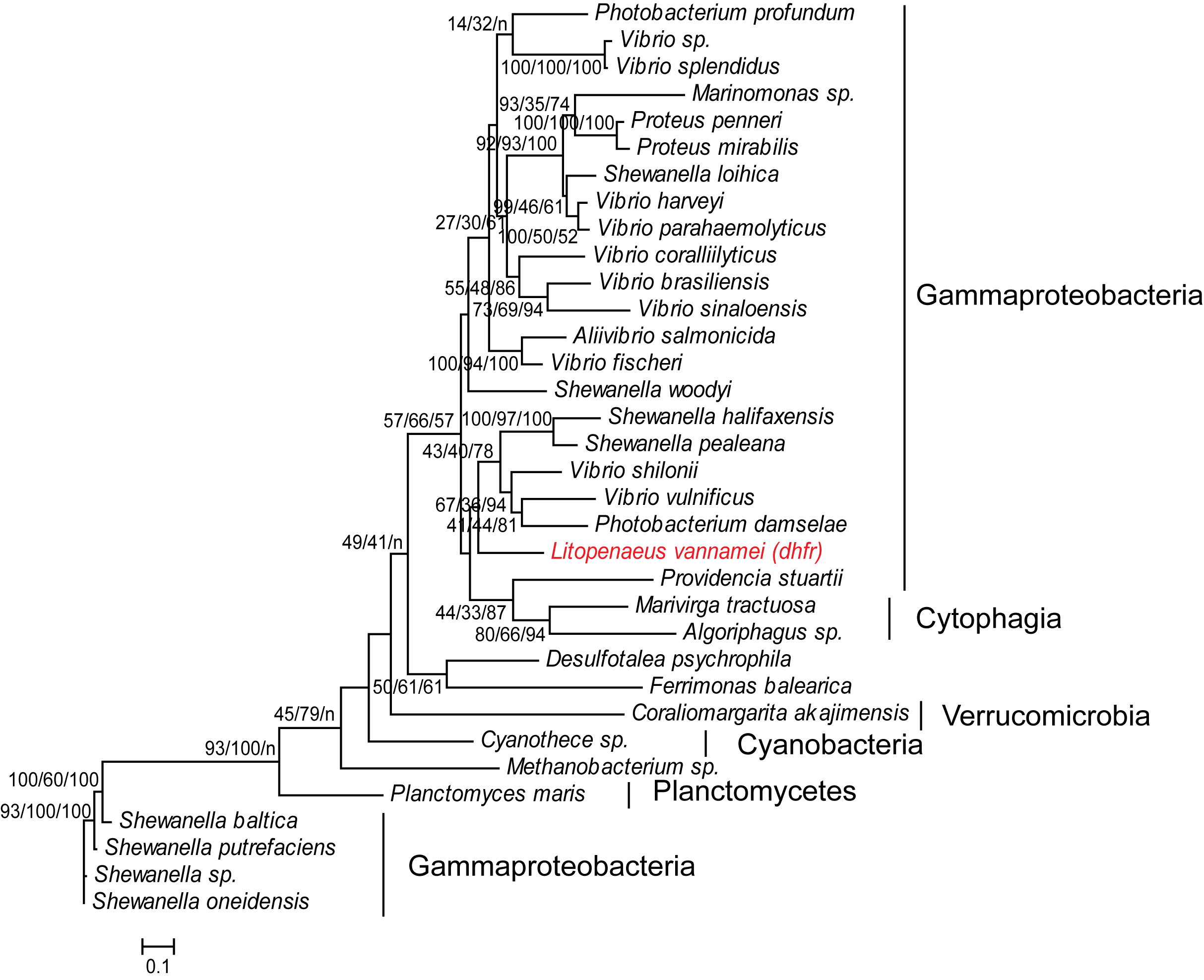
**

Figure S9. Phylogenetic tree of *dhfr* and its homologs. The support values of ML, NJ and BI analysis displayed beside each node.

**
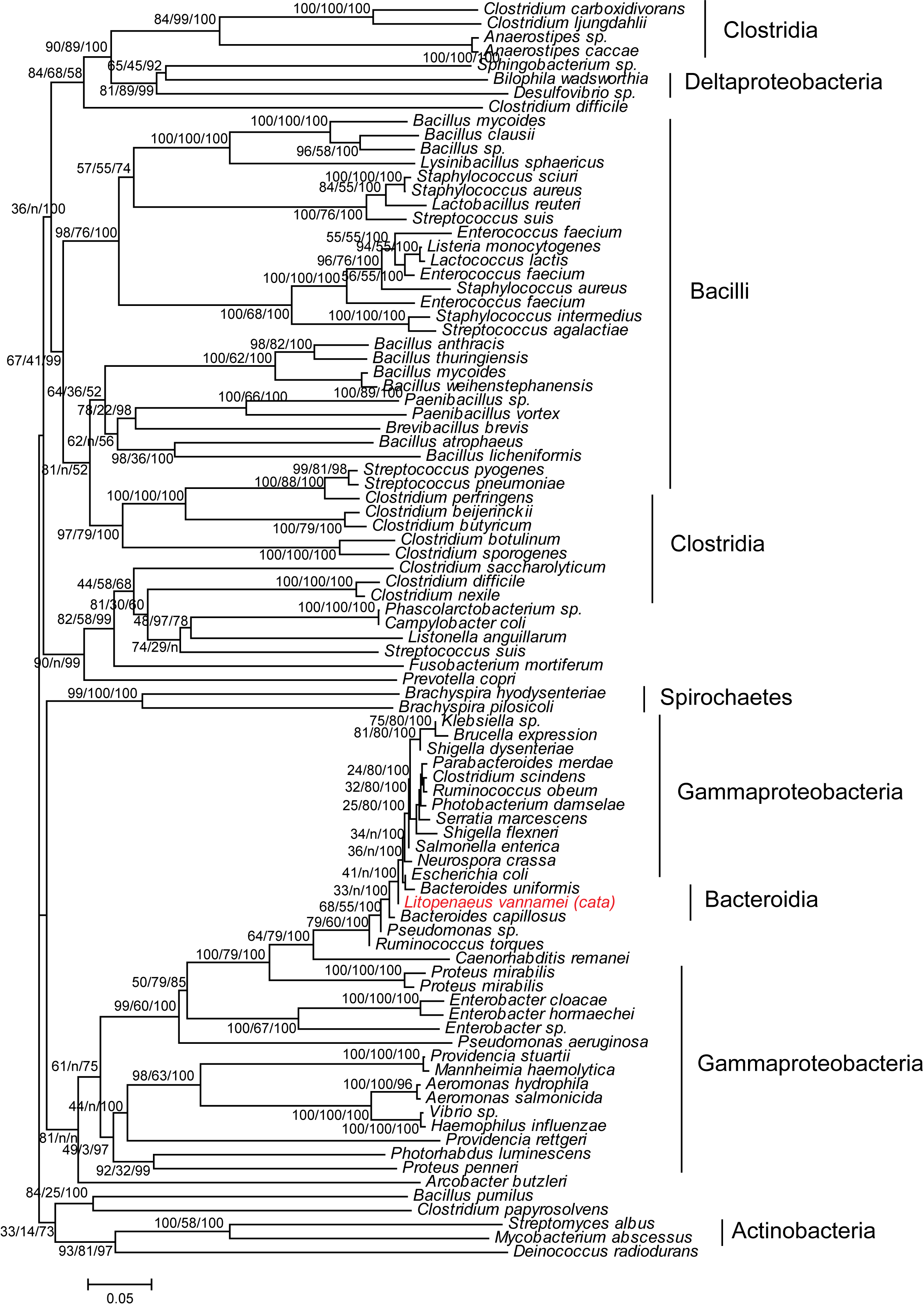
**

Figure S10. Phylogenetic tree of *cata* and its homologs. The support values of ML, NJ and BI analysis displayed beside each node.

**
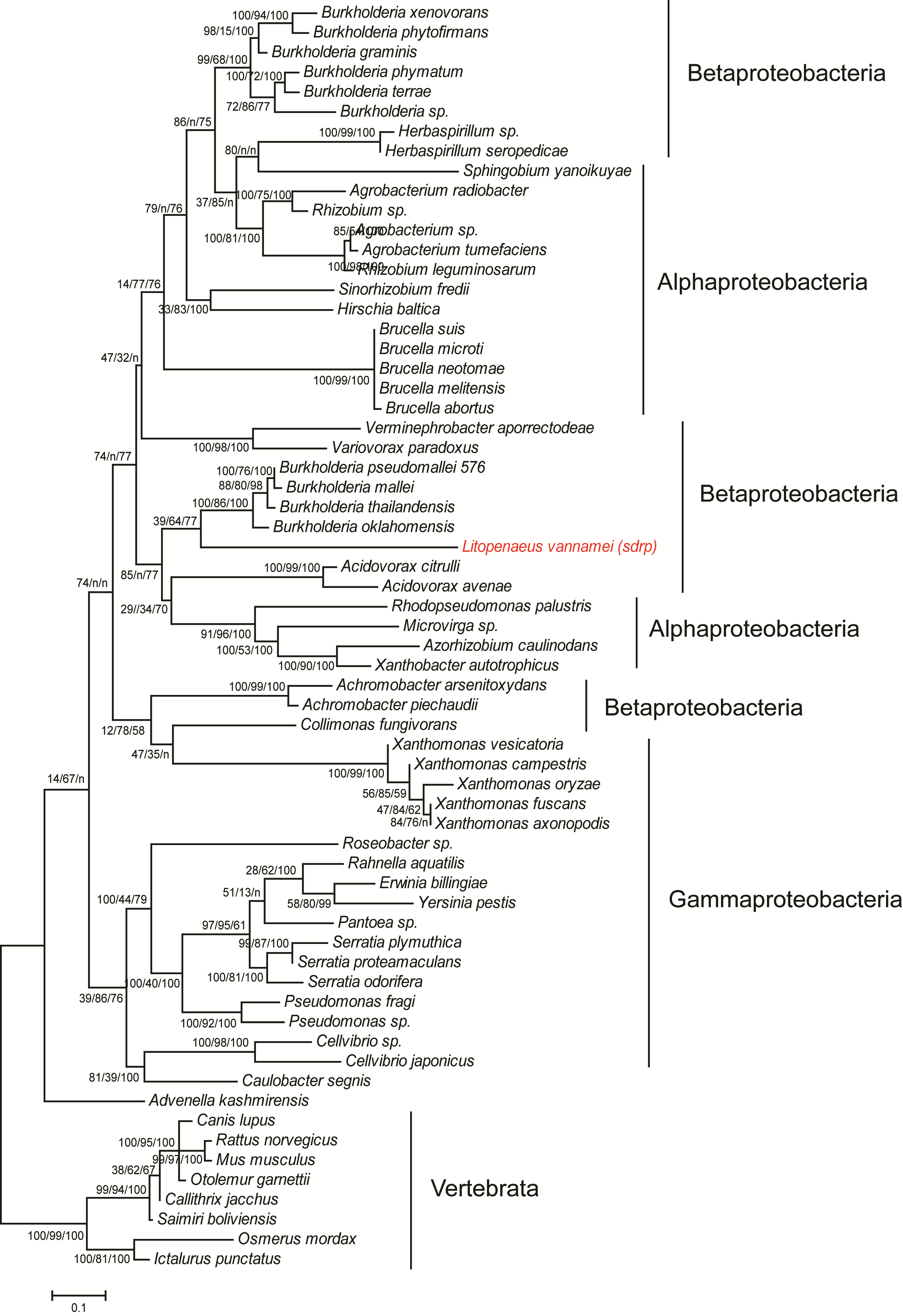
**

Figure S11. Phylogenetic tree of *sdrp* and its homologs. The support values of ML, NJ and BI analysis displayed beside each node.

**
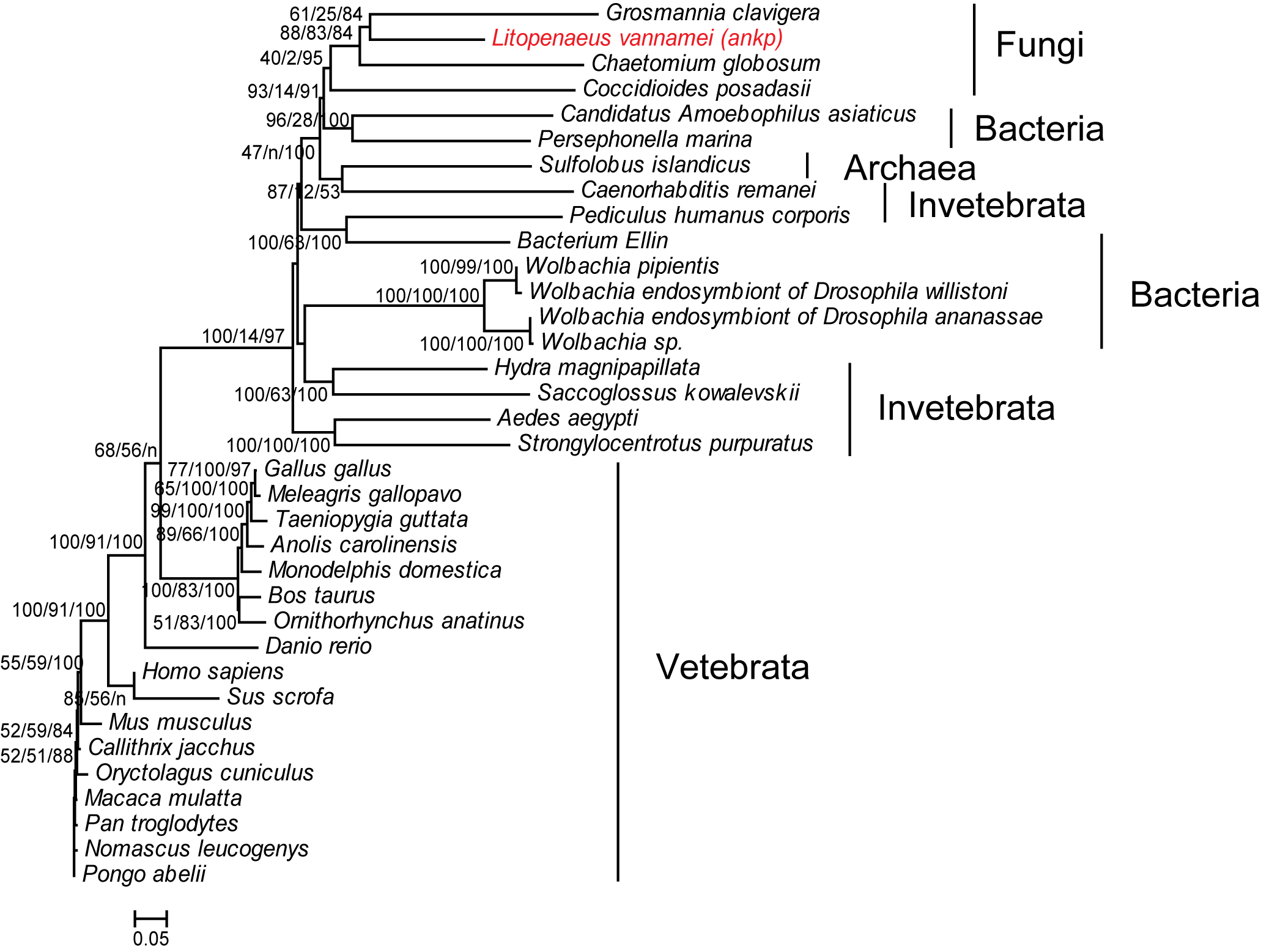
**

Figure S12. Phylogenetic tree of *ankp* and its homologs. The support values of ML, NJ and BI analysis displayed beside each node.

**
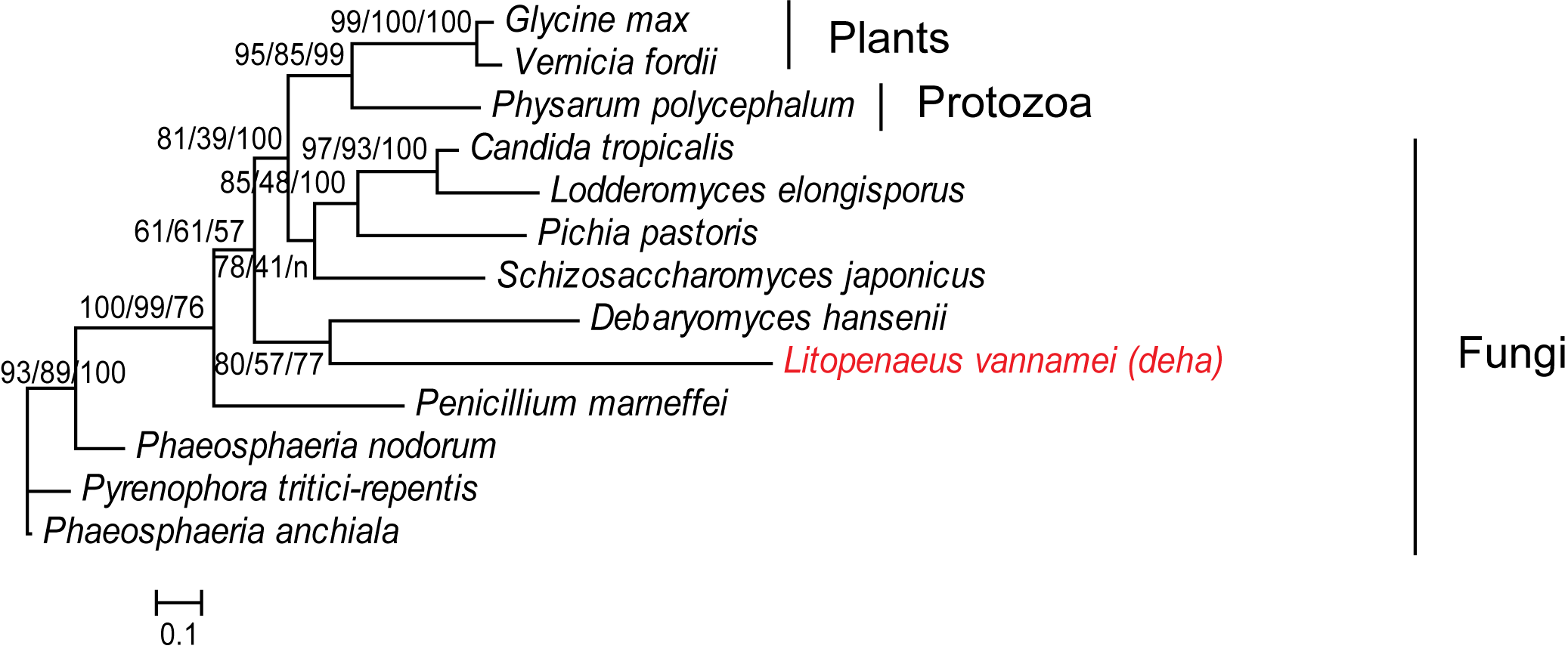
**

Figure S13. Phylogenetic tree of *deha* and its homologs. The support values of ML, NJ and BI analysis displayed beside each node.
